# A Web-Based Cancer Prevention Intervention for Rural Emerging Adults: Mixed Methods Development and Pilot-Testing Study

**DOI:** 10.2196/80803

**Published:** 2026-01-08

**Authors:** Echo L Warner, Alishia Kinsey, Barbara J Walkosz, Julia Berteletti, Kayla Nuss, Annelise Small, W Gill Woodall, Deanna Kepka, Douglas Taren, Meghan B Skiba, Dolores D Guest, Cindy K Blair, Judith S Gordon, David W Wetter, Evelinn A Borrayo, Kimberly L Henry, Andrew L Sussman, David B Buller

**Affiliations:** 1 College of Nursing, Huntsman Cancer Institute University of Utah Salt Lake City, UT United States; 2 Klein Buendel (United States) Golden United States; 3 Circle of Hope College of Nursing; Huntsman Cancer Institute University of Utah Salt Lake City, UT United States; 4 Department of Pediatrics Nutrition Center School of Medicine University of Colorado Anschutz Medical Campus Aurora United States; 5 College of Nursing University of Arizona Tucson, AZ United States; 6 Comprehensive Cancer Care Center University of New Mexico Albuquerque United States; 7 Department of Population Health Sciences, Huntsman Cancer Institute University of Utah Salt Lake City, UT United States; 8 University of Colorado Cancer Center Aurora United States; 9 Department of Psychology Colorado State University Fort Collins, CO United States

**Keywords:** emerging adult, web-based, intervention development, cancer, prevention, rural, community engagement

## Abstract

**Background:**

The rapid growth of user-generated web-based health information increases the complexity of cancer information seeking. One promising strategy for promoting high-quality cancer information consumption is through targeted interventions that are intentionally designed to reach individuals in the web-based spaces they occupy. However, there is a paucity of evidence-based information on the best strategies for designing and implementing web-based health behavior change interventions to improve individuals’ cancer-related knowledge and prevent cancer.

**Objective:**

This study aimed to develop and pilot test a theory-based intervention via the web to reduce 6 cancer risk factors among rural emerging adults (EAs) through community-engaged research.

**Methods:**

This mixed methods evaluation describes the development of a web-based cancer prevention intervention aimed at rural EAs aged 18-26 years in the United States and delivered in Facebook private groups. The intervention was guided by behavior change theory and cocreated with EA and Stakeholder Organization Advisory Boards to ensure relevance, accessibility, and appropriateness. We report on 3 formative surveys, a pilot intervention, protocol development, and the community-engaged process for intervention development. Descriptive statistics were applied to the surveys and pilot intervention baseline results to produce means and SDs using R.

**Results:**

We developed posts (n=400) for a Facebook feed aimed at reducing 6 cancer risk behaviors (unhealthy diet, lack of physical activity, tobacco use, alcohol use, sun exposure, and human papillomavirus infection) with iterative input from the EA and stakeholder advisory boards. Formative surveys with rural EAs (n=297) and a pilot study of the intervention with this population (n=26) were conducted. In the pilot study, the intervention reached participants across rural counties, with sustained engagement (post views=1060, reactions=346, comments=72) over a one-month period. Key modifications to the intervention content and design emerged from both advisory boards, the formative surveys, and the pilot intervention, focusing on using perceived reliable sources and direct links to source material.

**Conclusions:**

This web-based cancer prevention intervention is scalable and delivers engaging, evidence-informed health information to rural EAs. We offer key insights into the design and implementation of web-based cancer prevention interventions for EAs by describing the resources, timelines, and expertise needed to design and implement the intervention. Considerations for fully engaging EA and community stakeholder partners are presented, and we discuss how their involvement resulted in modifications that strengthened the intervention. Finally, we highlight the importance of theory-based health-behavior messaging, digital messaging skillsets, and platform-tailored dissemination strategies for maximizing web-based intervention acceptability.

**Trial Registration:**

ClinicalTrials.gov NCT05618158; https://classic.clinicaltrials.gov/ct2/show/NCT05618158

**International Registered Report Identifier (IRRID):**

RR2-10.2196/50392

## Introduction

Emerging adults (EAs) in rural and remote areas of the United States face unique challenges that increase their vulnerability to cancer. Emerging adulthood, a critical life stage from ages 18 to 26 years, is marked by increased autonomy and transitions in financial, residential, and employment responsibilities [1]. However, many EAs establish unhealthy lifestyle patterns during this period, including reduced physical activity, poor dietary habits, nicotine and tobacco use, binge drinking, sporadic sun safety practices, and skipping human papillomavirus (HPV) vaccination [1,2]. Living in a rural setting can heighten feelings of isolation and limit access to resources that support healthy behaviors [3]. These modifiable risk factors contribute to premature cancer morbidity and mortality, making rural EAs a priority population for cancer prevention efforts.

Given its popularity among EAs [4], using a social web-based intervention offers a promising avenue for addressing cancer prevention among EAs in rural areas. Over 80% of individuals in this age group access the internet several times per day [4]. Despite known flaws in information quality, web-based interventions that are strategically and theoretically designed are promising strategies for providing high-quality health information from trusted voices [5]. Web-based educational interventions can also disseminate timely and relevant public health messages, leverage user-generated content to personalize information, engage audiences in 2-way communication, and be used to detect and respond to emerging trends [6,7]. The success of web-based interventions hinges on their ability to address the broader social determinants of health, community and cultural perceptions, and built environments in which individuals live. However, web-based media can also perpetuate harmful and misleading health information [8-11], and interventions that relate accurate and truthful cancer prevention strategies are needed [12-16]. Studies of web-based interventions have often lacked theoretical grounding, rigorous design and evaluation procedures, and guidelines on the development of content, limiting the rigor and reproducibility of this intervention approach.

Rural communities often face unique barriers to engaging in healthy lifestyles, such as limited health care access, socioeconomic challenges, and geographic isolation [17-19]. EAs have limited interaction with traditional community channels like schools, workplaces, and health care settings, so web-based interventions may provide unique opportunities for tailored, real-time engagement with EAs. Use of community engagement strategies in the development of both the content and structure of social media interventions can increase relevance to the specific needs of rural EAs [20,21]. A community-engaged approach to web-based intervention design should increase the relevance of interventions to the specific needs of rural EAs.

In this context, we developed a theory-based, web intervention to reduce 6 cancer risk factors common among rural EAs, including physical inactivity, unhealthy diets, nicotine/tobacco use, binge drinking, unprotected ultraviolet exposure, and preventing HPV infection. It was designed and pilot tested using community-engaged methods. Herein, we describe the development, pilot-testing, and refinement of our intervention prior to its launch in a randomized quasi-experimental trial. This developmental research project involved community-engaged research methods with a dynamic group of EAs and community stakeholders, a theory-informed process for creating content tailored to rural EAs, and formative research to refine and pilot test the intervention.

## Methods

### Setting and Study Design

The overall aim of the study was to create a web-based intervention, named PEAK Wellness Chat, and evaluate its effectiveness in a sample of EAs in a stepped wedge randomized quasi-experimental trial design (NCT05618158) [22]. The private-group function in Facebook was the platform for intervention delivery. Facebook is used by a large number of American adults, regardless of race/ethnicity, including EAs (67% of adults use it and 70% of rural adults) [23]. The private-group function cultivates the privacy of group members, which is essential to control experimental exposure to the intervention and avoid experimental contamination. Facebook also allows a variety of content delivery by length and type (eg, images and videos as well as links to other sites and sharing of Facebook posts from other organizations) and allows posts to remain in the group in perpetuity. Facebook also records engagement of each participant with posts (ie, reactions and comments) and tracks retention via group membership, to enable testing of intervention dose effects. Finally, the Facebook algorithm promotes private-group posts in participants’ feeds in terms of frequency and prominence when they engage more with group posts.

### Ethical Considerations

This study was approved by the WCG Institutional Review Board (IRB20223673 and IRB20223673). Informed consent was obtained and documented electronically prior to enrollment in the formative surveys and pilot study described below. Privacy and confidentiality were maintained by storing all research subjects’ data in password-protected and encrypted drives. No identifying information is presented herein. Participants were provided with a modest incentive for completing the formative survey (US $40) or pilot study (US $50).

### Community-Engaged Strategy for Developing PEAK Wellness Chat

**Figure 1:**
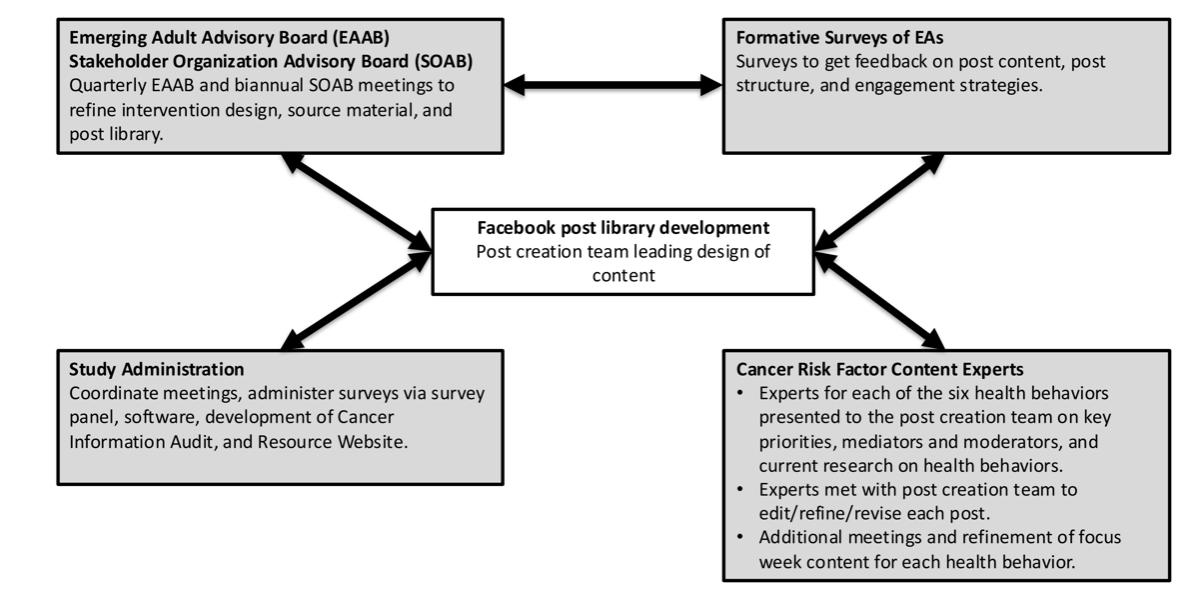
Community-engaged strategy for designing a Facebook post library. EA: emerging adult.

A library of Facebook posts for the PEAK Wellness Chat intervention was created by a core team of investigators and staff with expertise in cancer prevention, public health promotion, health communication, and emerging adulthood. The library was iteratively developed with structured community input from an Emerging Adult Advisory Board (EAAB) and Stakeholder Organization Advisory Board (SOAB), responses by EAs to formative surveys, and guidance by experts in the 6 cancer risk factors. Intervention management by the study’s administrative team and engagement by EAs were assessed in a pilot study (Figure 1). The administrative team supported the overall study procedures, meeting facilitation, coordination and deployment of surveys, recruitment efforts, and software acquisition for post illustrations. Additionally, the administrative team produced and maintained a cancer information audit and a resource website. The cancer information audit was updated weekly with emergent cancer information and news about health and wellness, alongside any emerging internet trends and current events that should be incorporated into posts to promote relevance and relatability of post content. The resource website contained additional information about the cancer risk factors, community and national resources (eg, quit smoking hotline and food banks), and links to health education resources.

### EAABs and SOABs

We used partnership processes aligned with the community-based participatory research model of Wallerstein et al [20] as a framework for the participatory approach. Through regular advisory board meetings, we addressed: (1) knowledge of rural “Contexts” that inform catchment area needs; (2) culturally informed “Partnership Processes” that facilitated a regular and iterative feedback cycle between the advisory boards, content experts, and post creation committee to refine the design features, language, and content of the Facebook posts; (3) development of responsive “Intervention & Research” protocols for rural conditions; and (4) participatory “Outcomes” that were iteratively disseminated to community partners through the intervention development and advisory board meetings (and are continuing during the intervention implementation in the trial).

### EAAB and SOAB Composition

A total of 15 EAs, representing the composition of participating rural communities, agreed to serve on the EAAB. EAAB members were recruited through word-of-mouth referrals from the research teams’ community networks. The EAAB held quarterly meetings to give input on the lived experiences of EAs, review the web-based intervention plans and protocols (eg, engagement strategies in posts), consider appropriateness, relevance, and engagement of proposed posts, and advise on recruiting EAs for the trial. Project investigators and staff at each participating Cancer Center also led direct engagement efforts with stakeholder organizations that serve rural EAs to convene members of the SOAB, which had 14 members. The SOAB complemented the EAAB by identifying local resources for cancer risk reduction, reviewing intervention plans, protocols, and posts, and supporting recruitment and dissemination efforts in biannual meetings. Topics for discussion in the EAAB and SOAB meetings were jointly decided based on project needs, PEAK Wellness Chat content, and priorities of the board members.

### Creation of the PEAK Wellness Post Library

The library of Facebook posts contained messages educating EAs about strategies to improve the 6 cancer risk behaviors. Messages were also created to increase EAs’ media education about cancer and cancer prevention. Finally, posts were produced to enhance EAs’ skills for communicating with family and friends because they reported: (1) living in a variety of arrangements, (2) relying on family and friends for acquiring and preparing food and paying for health care, and (3) living with others who engaged in the cancer risk behaviors. A focus week of up to 8 posts for each cancer risk factor was designed to emphasize messages on one important principle for improving the cancer risk factor. A focus week of posts on each of the 6 risk factors is being delivered during each 3-month period during the intervention.

### Post Development Process

Each post was designed to address key features of health behavior change theories (self-determination theory, social cognitive theory, and diffusion of innovation theory [24-26]) to ensure the posts are conceptually driven to impact EAs’ health behaviors. Specific principles incorporated into the posts included: intrinsic motivation, extrinsic motivation, social support, relatedness, personal goals, ability to control, self-efficacy, response efficacy, injunctive norms, descriptive norms, cancer risk perceptions, response cost, compatibility with values, observable benefits, autonomy, and simplicity. Table S1 in Multimedia Appendix 1 provides examples of posts incorporating each principle. Principles from self-determination theory, social cognitive theory, and diffusion of innovation were operationalized using messaging design and engagement strategies to ensure messages were not only informative but also encouraged motivation and engagement. Message design strategies included polls, narratives, calls for sharing, testimonials from EAs, source credibility, behavioral skills, and referrals to community resources. Engagement techniques included content from near peers, invitations to comment, cultural barriers and facilitators, images of ethnically diverse EAs, stories, videos or other visuals, question and answer formatting, and behavioral change techniques (ie, self-improvement and freedom to act), and high-interest, useful, and current event content. The framework also included message strategies to promote engagement (eg, visuals, current event content, cultural barriers/facilitators, polls, requests for comments, story requests, knowledge test, picture request). These features will be tracked and evaluated to ensure a variety of engagement strategies and best practices for health behavior change are integrated throughout the campaign. The post library was created and maintained in Microsoft Excel, where each post was coded for key theoretical concepts and engagement features (Table 1).

For this phase of intervention development, the EAAB and SOAB provided timely feedback on the content and design of posts, novel engagement strategies (ie, videos, use of polls), trustworthy and credible source material, and incorporation of local resources where possible. Along with the post library, the authors developed protocols for disseminating the intervention, moderating the Facebook group, and verifying the legitimacy of a participant’s Facebook account. These included procedures for scheduling posts, responding to questions, and auditing the cancer prevention communication environment to identify emergent topics that should be included in the intervention to make messages relatable, relevant, and engaging. For example, the protocols included specific guidance for the moderator. Per the protocol, the moderator reacted (ie, like, love, and care) to every participant’s comment left on posts. If a participant’s comment resonated with the moderator, they would respond with their own comment to foster an authentic sense of community. Moderators will also respond to any direct Facebook messages participants send regarding the study (ie, questions about certain topics the participant does not want to share publicly). The protocol also includes boundaries for the moderator to model feasible and replicable interactions with the groups (eg, not to like comments that are misinformation).

**Table 1:** PEAK wellness post library.

Post features	Definition	Example
Post #	Indicates the order of the post and record number	1, 2, and 3
Date and time posted	Calendar date, time posted	26 February, 2025 9:00 AM
Day of week	Differentiate weekdays from weekends	Wednesday and Saturday
Topic	Identifies one of the 6 cancer prevention risk factors, media literacy, or family communication	Sun safety, HPV^a^ prevention strategies, healthy diet
Message	Main content shared in the post	Knowing how to correctly use sunscreen to protect your skin is important. Here are some general guidelines.
Link	Source content link to be shared in the comments with viewers	https://www.cdc.gov/tobacco/campaign/tips/quit-smoking/index.html
Post visual suggestions	Indicates the type of visual content that should be included in the post	Team created/external content creator, TikTok video, YouTube video, infographic, or photo
Facebook post link	Link to the post on Facebook for tracking and review purposes	URL
Gender presentation	Indicate the visual gender presentation of people in the posts	Woman, man, N/A^b^ if none
Character features	Indicate the body type, presenting race/ethnicity, and conventionally attractive features	Athletic build, Asian, conventionally attractive
Colors used in graphics	2-3 main colors	Red, blue, green
Engagement post	Indicator of whether or not the post is designed to promote engagement	Poll, “Tell us in the comments,” share a story, knowledge test, picture request
Primary/secondary outcome addressed	Health behavior addressed with the post, also includes key topics addressed in the post	Cancer risk, autonomy, cooking skills, increased physical activity, preventing drunk driving, vaping knowledge, winter sun protection
Theoretic mediators	Key behavior mediators designed to promote behavior and health belief change	Intrinsic motivation, extrinsic motivation, social support, relatedness, personal goals, ability to control, self-efficacy, response efficacy, injunctive norms, descriptive norms, cancer risk perceptions, response cost, compatibility with values, observable benefits, simplicity
Message design features	Key design features based on health communication practices	Moderator instructions, referral to community resources, testimonials from EAs^c^
Engagement techniques	Strategies used in the post to promote engagement	Visuals, current event content, cultural barriers/facilitators, poll, “Tell us in the comments,” share a story, knowledge test, picture request

^a^HPV: human papillomavirus.

^b^N/A: not applicable.

^c^EA: emerging adult.

### Formative Surveys With Rural EAs

#### Overview

During the development of the post library, we administered 3 web-based surveys with rural EAs to evaluate initial reactions to the posts (Table 2). The surveys also collected information on EAs lived experiences, use of Facebook and other web-based channels, and perceived credibility of potential information sources used in the posts.

**Table 2:** PEAK Wellness Chat intervention development surveys.

Survey name	Purpose	Sample size (N)	Fielded dates
Formative survey 1	Health topics of interest, popular influencers or content creators, and credibility of health information sources.	100	February 2024
Formative survey 2	Discussing health behaviors with others, cancer risk behaviors by household, Facebook group name, and dietary experiences	100	July 2024
Formative survey 3	EAs’^a^ engagement in physically active jobs and recreational activity, access to and perceptions of healthy and affordable food	97	January 2025
Pilot pretest survey	Baseline test of health behaviors, mediators, other covariates, and demographics	26	March 2024
Pilot posttest survey	Baseline test of health behaviors, mediators, other covariates, and demographics	23	April 2024

^a^EA: emerging adult.

#### Participants

Eligible individuals were ages 18-26 years, living in counties designated as Rural Urban Continuum Codes (RUCC) 4-9 [27] in Colorado, Idaho, Montana, Nevada, New Mexico, Utah, and Wyoming, and Rural Urban Commuting Area Codes 4-10, which classifies rurality by census tract [28] in Arizona where RUCC codes excluded several key rural communities. To facilitate adequate recruitment for the full trial, we expanded our recruitment to states beyond the original 4 corners states, and this modification was reflected in the formative survey participants. They also needed to be able to access a web-based survey and to use Facebook once or more times per week. Participants were identified through a survey panel company, Dynata [29]. The overall survey response rate was 302/1080 (28.0%), and the overall survey completion rate was 297/302 (98.3%).

#### Measures and Analysis

Posts were purposefully selected for inclusion in the surveys, focusing on issues that arose during post development and discussions with the EAAB, SOAB, and content experts. For example, the credibility of sources cited in the posts was a concern, so we selected posts with local and national source material (eg, Centers for Disease Control, newspapers, and local health departments). Respondents were asked to (1) rate each post for appropriateness, relevance, believability, amount of text, and trustworthiness on 5-point Likert scales; (2) indicate their potential engagement with the post through reading, scrolling past, reacting to, commenting on, and clicking on links (no, yes maybe, yes definitely); and (3) suggest ways for improving the post (open ended).

Surveys also contained questions on EAs lived experiences that informed postdevelopment. In the first survey (February 2024), participants were asked about topics of interest, popular influencers/content creators, and the credibility of information sources. In the second survey (July 2024), they were reported on discussing health behaviors with parents, siblings, partners, and friends, cancer risk behaviors by household members (use of nicotine products, consumption of alcohol, and intentional suntanning), preferences for the Facebook group name, and dietary experiences including sources of food, dependence on others for food purchasing, and sources of free food. In the third survey (January 2025), questions inquired about EAs’ engagement in physically active jobs and recreational physical activity outside work, access to healthy and affordable food, and perceptions about healthy eating. Formative surveys are available upon reasonable request from the authors.

#### Analysis of EA Survey Responses

Summary statistics of participants’ sociodemographics, lived experiences, and post feedback were calculated for each survey, using RStudio (Posit Software, PBC), 2024. Two open-ended questions (“What would you comment on this post?” and “How would you improve this post?”) were coded to identify emergent themes. All responses were categorized into an individual theme. From the Comment question, 134 responses were analyzed, and 53 responses were excluded due to being out of context, incomplete statements, or illegible responses. The 81 responses coded resulted in the following themes: message-specific reactions and affirmative comments. From the Improvement question, 506 responses were analyzed, and 73 responses were excluded due to being out of context, incomplete statements, or illegible responses. The remaining 433 responses were coded by 2 coders in the following themes: suggestions on image, dislike or wrong audience, more information requested, reinforce source/suggestions on source, suggestion on content, too much information, unclear message, nothing to change, unsure.

Survey and open-ended feedback were iteratively reviewed by the full study team to improve the content and format of the posts in the intervention feed and ensure that posts were responsive, timely, and engaging for rural EAs. Summary reports of formative survey results were discussed in EAAB and SOAB meetings to review thematic findings and explore contextual and social considerations.

### PEAK Wellness Chat Pilot Study

#### Overview

A 4-week pilot test of the intervention was conducted with a sample of rural EAs to refine procedures for the full trial pertaining to recruitment, baseline and posttest surveys, retention, and intervention protocols. Data were also obtained to confirm that posts were engaging for EAs. The pretest survey is available as a Multimedia Appendix 2.

#### Participants

Participants were recruited by Verasight [30], a research services company that recruits participants for studies through a web-based panel and advertising. Eligible participants for the pilot study were ages 18-26 years, living in counties designated as RUCC 4-9 in Arizona, Colorado, New Mexico, and Utah, and able to access the web-based survey. Pregnant participants were excluded because they may depart from their normal dietary and activity levels and consumption of alcohol and nicotine due to the pregnancy. Participants were screened for having an existing Facebook account for at least 1 year with weekly activity, with 2 EAs deemed ineligible. In addition, 3 EAs were excluded because they did not friend the Group Moderator and could not be joined to the Facebook private group for the pilot feed.

#### Pilot Study Procedures

Participants were enrolled in a single group, pretest-posttest design. Initially, they completed a baseline survey in REDCap (Research Electronic Data Capture; Vanderbilt University). The intervention was presented in a Facebook private group, with 2 posts made per day. During a 7-day period in the month, 8 posts in a focus week on physical activity were posted. This focus week strategy was designed to provide an in-depth intervention on improving self- and response-efficacy, increasing perceived risk associated with not being physically active [31-34], and linking cancer prevention to personal physical activity goals. During the 4-week campaign, 62 posts were made in the private group feed across domains related to physical activity (n=13), diet (n=7), sun safety (n=7), tobacco cessation (n=6), alcohol reduction (n=7), HPV prevention (n=7), media education (n=3), and family communication (n=2). Media literacy and family communication content were more general and not specifically related to the 6 health behaviors. At the end of the 4-week intervention period, participants were invited to complete a posttest survey in REDCap.

#### Measures and Analysis

The pretest and posttest surveys included questions about the 6 cancer risk factors: physical activity (Global Physical Activity Questionnaire [35]), diet (dietary screener; meal behaviors, food insecurity) [36,37], alcohol intake (Alcohol Use Disorders Identification Test–Consumption [38]), nicotine product use (30-day and 7-day smoking or vaping, quit ladder) [39,40], HPV prevention (initiation and completion of multi-shot vaccine series) [41], and ultraviolet protection (use of personal sun protection practices and sunburn) [42-45]. In addition, questions assessed basic needs, self-efficacy for cancer risk-reduction behaviors, cancer information overload, digital media use, health insurance coverage, last routine check-up, personal and family cancer history, and demographics.

Behavioral and experiential engagement with posts in the Facebook private group feed was measured. Behavioral engagement was assessed in two ways: (1) project staff kept a log of posts published to the Facebook private group that tracked date and time posted, topic, and post visual (graphic, gif, image, link, poll, or video); and (2) project staff extracted Facebook metadata reported in the private group platform, which included obtaining the total number of views, likes (eg, like and sad), and comments (comments from participants, comments from moderator) [46]. Facebook’s reporting function permitted likes and comments but not views to be associated with specific users. The posttest survey contained questions assessing participants’ experiential engagement with the intervention (ie, frequency of reading posts and sharing of content with others). Given the very small sample, the analysis involved descriptive statistics. All analyses were conducted in RStudio (version 4.4.2; Posit Software, PBC). The STROBE (Strengthening the Reporting of Observational Studies in Epidemiology) checklist is provided in Multimedia Appendix 3.

## Results

### Facebook Post Library

**Figure 2:**
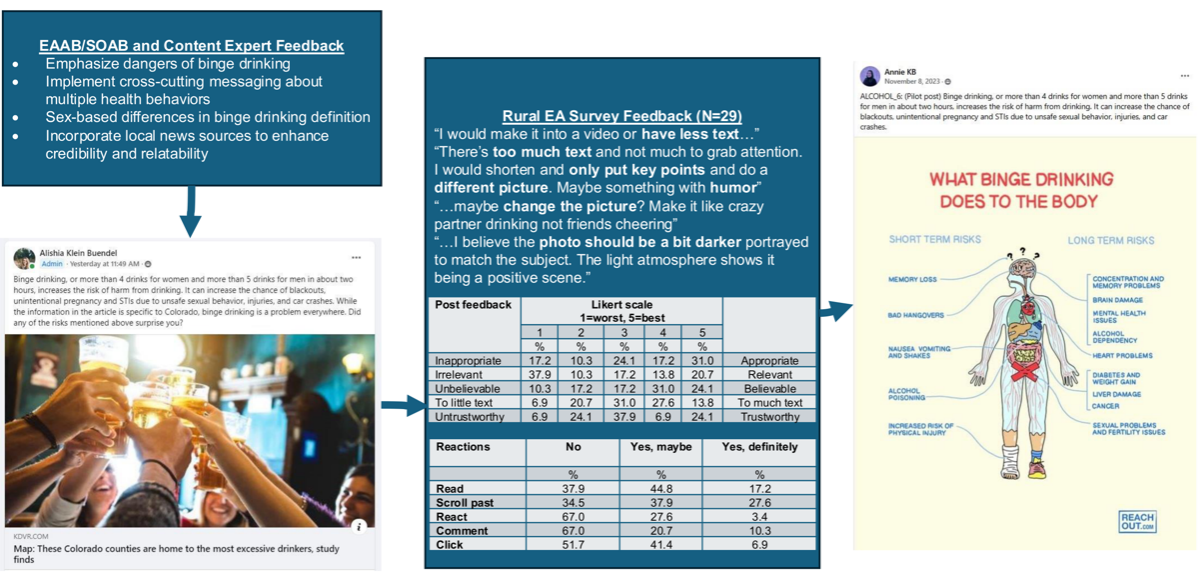
Evolution of a post through an iterative community-engaged development process. EA: emerging adult; EAAB: Emerging Adult Advisory Board; SOAB: Stakeholder Organization Advisory Board.

We produced a library of 400 Facebook posts on reducing the 6 cancer risk behaviors, media education, and family communication, using a real-time agile process to ensure posts engaged EAs. The development process followed an iterative feedback loop for each new post created. Content experts identified the key topics and information to address in the posts and essential mediators of behavior change for the cancer risk behaviors. The content from these presentations was adapted by a group of investigators and project staff into posts following the theoretical framework shown in Table 1. Content experts reviewed and approved the posts for inclusion in the final PEAK Wellness library, suggesting revisions that were incorporated to finalize the posts. Selected posts were pretested by having them reviewed by both the EAAB and SOAB, and included in the formative surveys for feedback as needed before finalization. Figure 2 illustrates the evolution of one post (about binge drinking) that was created using a photo visual and a local news source.

### Formative Rural EA Surveys

Formative surveys with rural EAs provided insights about developing, revising, and finalizing posts, health behavior topics of importance, and contextual information to include to improve the posts. The 3 formative surveys with EAs obtained responses from 99, 98, and 100 EAs, for a total sample of 297 rural EA respondents. As shown in Table 3, the average age of respondents was 22.5 (SD 2.49) years. The sample of participants was primarily identified as 72.4% (n=215) female, 24.2% (n=72) Hispanic, 70.7% (n=210) White, 54.5% (n=54) high school educated or less, 43.2% (n=82) unemployed, 46.2% (n=90) living with a spouse/partner, and 34.3% (n=99) living with other relatives besides parents. The majority, 63.6% (n=63), had health insurance, and many EAs involved others in their health decisions, primarily parents (56/99, 55.6%) and a spouse/partner (40/99, 40.4%). Respondents resided in 8 states, with subsamples ranging from Nevada (26/297, 8.8%) to Idaho (61/297, 20.5%). Across the 3 formative surveys, respondents provided feedback about 61 posts (survey 1=21 posts, survey 2=20 posts, survey 3=20 posts).

Content analysis of the 2 open-ended questions asking about how participants would engage with the posts and improve the posts resulted in 2 themes for post comments and 9 themes for improving posts (Table 4). Content analysis was applied by one research team member, who met with 2 other researchers to discuss definitions, interpretations, and the application of codes. Discrepancies were resolved through consensus, after which coding was finalized. In terms of comments, themes focused on respondents’ initial reaction to the post content and affirmations or approvals of the post content, creator, or message. Most posts received overwhelmingly positive feedback, but there were suggestions for improvements on visual appeal and less text across many posts. Due to this feedback, the post campaign library was revised to incorporate more video and photo content and eliminate text-only posts. Source material was a primary area of feedback that also resulted in changes to the campaign library, to provide greater transparency in primary source material and emphasize trusted organizations (local newspapers had lower levels of trust, while national organizations were seen as more trustworthy). Another unique theme related to the relevancy of the content, with sizable numbers of rural EAs reporting that they did not drink alcohol and thus felt alcohol-related posts were irrelevant. This reinforced the decision to focus the intervention on all 6 cancer risk behaviors rather than just one, so participants would likely receive at least some posts relevant to them. A final salient theme was the need for relatable characters in the posts. This request led us to partner with EAAB members and content experts to identify EAs who volunteered to record videos in which they talked about their experiences with each health behavior. These videos were developed following best practices for health communication via microvideos [47-49], with scripting designed to concisely convey the most important and relevant information and be emotionally engaging and authentic. Revised posts were reviewed by the health behavior content experts to ensure information accuracy.

**Table 3:** Formative rural emerging adult surveys respondent demographics.

Category		Total (N=297)
Age (years) (n=297), mean (SD)		22.5 (2.49)
**State of residence (n=297), n (%)**		
	Arizona	29 (9.8)
	Colorado	34 (11.4)
	Idaho	61 (20.5)
	Montana	37 (12.5)
	New Mexico	48 (16.2)
	Nevada	26 (8.8)
	Utah	32 (10.8)
	Wyoming	30 (10.1)
**Gender identity (n=297), n (%)**		
	Women	215 (72.4)
	Men	70 (23.6)
	Transgender/gender fluid/nonbinary	11 (3.7)
**Ethnicity (n=297), n (%)**		
	Not Hispanic/Latino	216 (72.7)
	Hispanic/Latino	72 (24.2)
**Race^a^ (n=297), n (%)**		
	American Indian/Alaska Native	26 (8.8)
	Asian	4 (1.3)
	Black/African American	17 (5.7)
	White	210 (70.7)
	More than one group	19 (6.4)
**Employment^a,b^ (n=197), n (%)**		
	Full-time	66 (38.1)
	Part-time	49 (24.1)
	Looking for work	37 (13.1)
	Unemployed, caregiver, student	82 (43.2)
**Living situation^b^ (n=197), n (%)**		
	Alone	20 (13.0)
	With parents	57 (22.2)
	With roommates/friends	27 (14.1)
	With spouse/partner	90 (46.2)
	With other relatives	99 (34.3)
**Education** ^c^ **(n=99), n (%)**		
	High school or less	54 (54.5)
	Some college/trade school	17 (17.2)
	Four-year college degree	20 (20.2)
	Graduate degree	6 (6.1)
**Health insurance^c^ (n=99), n (%)**		
	Yes	63 (63.6)
	No	15 (15.2)
**Who helps with health decisions?^a,c^ (n=99), n (%)**		
	I make them alone	14 (14.1)
	Parents	56 (55.6)
	Roommates/friends	10 (10.1)
	Spouse/partner	40 (40.4)
	Others	15 (15.2)

^a^Multiple responses could be selected, so totals may not equal 100%.

^b^Question not asked in the third round of surveys.

^c^Question not asked in the second or third round of surveys.

**Table 4:** Thematic categories of feedback with reactions and recommendations for posts by rural emerging adults (EAs) responding to the formative surveys (N=297), Emerging Adult Advisory Board (EAAB) members (N=17), and Stakeholder Organization Advisory Board (SOAB) members (N=16).

Theme	Definition	Example quote on how to improve posts	Resultant modifications
**Comment**			
Message-specific reaction	Unique specific reactions, opinions, or inquiries about the message or content.	What product do you use to clear up skin? Groceries are very expensive nowadays, you can just go shopping and take whatever you want. You need to think twice… Wow, I didn’t know this! Maybe I should try this. How urgently should I get it [HPV vaccine]?	Specific unclear messages were revised; additional information was provided
Affirmative comment	Positive receptivity or relatability, makes a direct affirmative comment, associates a positive reflection, or indicates a positive personal resonance on the message, content, or creator.	That this statement is true That i like the message Thank you for trying to help society How helpful this ad would be This is great…how things like this can help people I appreciate the vote [of] confidence How I can relate	Prioritization of posts that received ample affirmations
**Improvement**			
Suggestions on image	Improving visual elements that would enhance the overall impact and reach of the posts. Selecting images that are, but not limited to, authentic, relevant to the topic, visually appealing, and tailored to look like and engage the intended audience effectively.	Using a real image as opposed to clip-art/AI art style image will help, as I would immediately see this as an ad and scroll past it. Also, the tips at the top make the block of text too large, leading to less people reading it. Change the stock photo to a clearer image, this one is a bit blurry Since it is geared towards young adults, I would change the picture to be of a young adult rather than an older woman.	Inclusion of more realistic images, improvements in image resolution, and emphasis on younger appearing characters in posts
Dislike or wrong audience	The content, although potentially valuable or well-constructed, fails to engage them personally; misalignment with their interests; direct or indirect discontent with the post; lack of connection with the message or intent.	I’m sure it’s helpful to many people but for me it isn’t It's a great post just not for me because I don't drink. It is well made, just irrelevant to me. I don't really keep up with influencer content, I don't know why I should stop and listen or care about this person’s experience with quitting	Revision of posts deemed irrelevant
More information requested	Content, while engaging, lacks adequate detail or depth.	I would just add more facts of why you overeat when you’re watching a show because it can happen More facts to back up the information Give a description about what HPV is for people who do not know and how one might contract this	Revision of posts to include additional detail where possible; additional posts created when more in-depth information was requested
Reinforce source or suggestions on source	Demands for greater transparency and verification concerning the origins or credibility of the information presented in post; any suggestion about the source materials or the need to further elaborate on them; avoid certain sources due to local political climate, testimonials must seem trustworthy, such as those from partnering with influencers.	Make sure any links provided and information listed is factual and from a trusted source. The “doctor” needs to have credentials posted so he is more believable. Anyone can say they are a doctor. I would add sources that you could click on so you could read on more about it Maybe linking sites to help people quit We are just a very politically charged town, I know some will see CNN and just avoid it	Inclusion of source material from various outlets and improved transparency of source material across posts
Suggestions on content	Direct and postspecific suggestions that reflect a collection of feedback aimed at improving the effectiveness or engagement; recommendations on, but not limited to, enriching the informational depth, avoid use of scare tactics, shocking words, or condescension, keep tone positive, optimizing visual appeal, adding interactive content, and content’s relevance and accuracy.	Add subtitles I would tell a story to help build common ground with people who are addicted Maybe get a more attractive guy have him lose the hat, people stop scrolling for people pleasing to the eye Explain why quitting nicotine is hard and not to beat yourself up if you can’t quit immediately If she was wearing reflective clothing it'd get the point across better. I would show examples of what could happen, perhaps provide stories from real life people as well I’d be more likely to click on a poll post to also see how other people responded	Subtitles added to video posts, emphasis on images that align with main messages, increased use of interactive posts like polls, addition of posts with real-world rural EAs for relatability
Too much information presented	Concerns about the overload of text or information, highlights the need for simplification and streamlining in content presentation, emphasizing that concise and clear communication often resonates better where attention spans are short. Brief posts and videos are preferred.	I would make it a little more less detailed Maybe make the video a little shorter, or have graphic on the screen with the overarching message (like the power of new habits or something) This post is direct and straight to the point. It does have a lot of text, so I would find a way to shorten the text to make it more enticing to read. Limit what is said before the post. I've recently looked up the information it is talking about but it says a bit too much before having to click the link.	Revision of posts to reduce text, shorten videos (limited to under 2 minutes), and abbreviate post captions
Unclear message	Content’s intended message is not comprehensible or straightforward, leading to confusion and diminished engagement.	I couldn't really tell if it was about limiting screen time or food intake, and didn't really get how it was connecting them, so maybe make the overall message/goal clearer Use less biased language, as it is already painting these sponsorships in a bad light, which will affect who clicks on the link more than a neutral factual title Starting with something beside the question, or maybe a different question. It just didn’t feel particularly intriguing. Make the message more clear. I do not really understand this video	Unclear messages were revised and reviewed with EAAB and SOAB members and content experts to improve clarity in messaging

### EAAB and SOAB Feedback and Resultant Modifications

Finally, feedback from the EAAB and SOAB largely focused on using widely recognized sources instead of smaller, more local sources for posts due to the fact that EAAB members stated they would feel weary of information provided by a source they did not recognize and providing links to every source used in the messaging as EAAB members felt the need to vet the information for themselves in order to trust it (Table 4).

### PEAK Wellness Pilot Study

There were 26 EAs who completed the pretest survey and took part in the pilot study. Three participants were lost to follow-up at posttest, with 23 (89%) participants completing posttests. Participants were 85% female and 35% Hispanic, with a median age of 23 years (SD 2.32). At pretest, many EAs reported having elevated cancer risk factors: 53% engaged in <150 minutes of moderate-to-vigorous physical activity weekly, 85% had low daily intake of fruits and vegetables, 35% used nicotine products, 58% had binged alcoholic beverages in the past 30 days, 65% were sunburned in the past 3 months, and 38% had not received the HPV vaccination.

Among all participants there were 1060 post views (mean per post: 16.6, mean per participant: 40.8), 346 reactions (mean per post: 5.4, mean per participant: 13.3), and 72 comments (mean per post: 1.2, mean per participant: 3.0). The participants viewed most posts and reacted to some posts (Table 5). Comments from participants on the posts included requests for more information, and the intervention manager posted 6 responses. EAs viewed posts on all topics a similar number of times, with most posts receiving 16-17 views. Posts on nicotine product use, alcohol consumption, diet, and physical activity received the most reactions (Table 6). The higher viewership of the physical activity posts was due in part to being the topic for the one focus week of posts in the pilot feed. Media education posts received the most comments. Most participants reported reading Facebook posts from private groups in general at least once or more per day, increasing from 58% at pretest to 77% at posttest. Feedback about the Facebook group was largely positive: at posttest 83% strongly agreed or agreed they would like to use the group, thought it was easy to use (91%), felt confident using the group (82%), and thought it was user-friendly (91.7% said it was good, excellent, or best imaginable, Table S2 in Multimedia Appendix 1).

**Table 5:** Aggregated feedback on posts reviewed in the formative surveys (64 posts, across 26 participants).

Engagement	Total	Mean per post	Mean per participant
Views	1060	16.6	40.8
Reactions (eg, likes)	346	5.4	13.3
Comments	72	1.2	3.0

**Table 6:** Aggregated engagement on posts reviewed in the formative surveys (64 posts, across 26 participants).

Post topic	Number of posts	Views, N (mean/post)	Reactions, N (mean/post)	Comments, N (mean/post)
Physical activity	13	216 (16.6)	77 (5.9)	14 (1.1)
Diet	7	118 (16.9)	44 (6.3)	10 (1.4)
Sun safety	7	115 (16.4)	30 (4.3)	8 (1.1)
Tobacco cessation	6	99 (16.5)	56 (9.3)	2 (0.3)
Alcohol reduction	7	125 (17.9)	52 (7.4)	5 (0.7)
HPV^a^ vaccination	7	115 (16.4)	26 (3.7)	4 (0.6)
Media education	3	52 (17.3)	15 (5.0)	8 (2.7)
Family communication	2	28 (14.0)	2 (1.0)	0 (0.0)

^a^HPV: human papillomavirus.

## Discussion

### Principal Findings

The purpose of this paper was to illustrate the systematic, iterative process for developing and pilot-testing a theory-based, web intervention that engages rural EAs and promotes healthy behaviors for cancer prevention in the United States. The process described herein fills a gap in the existing literature on realistic timelines, resources, and staffing required to rigorously design a theory-based web intervention by including practical examples, clear iterative steps in developing the intervention content, and community engagement strategies. We demonstrated how to combine health behavior theory, feedback from EAAB and SOAB advisory boards, and web design to develop a robust health intervention. Below, we describe keys to success, lessons learned (Textbox 1), and limitations of this approach to add to the literature on how to rigorously design reproducible web-based interventions about healthy behaviors.

**Lessons learned in developing a social media–based health education intervention.**

- Staffing and stakeholders: Coalescing and maintaining stakeholders required cultivating networks via dedicated personnel effort, and the strongest stakeholder engagement resulted from continuous quarterly engagement.
- Post creation: Developing theory-informed, community-engaged content for posts required an iterative design and revision process that included the community with stakeholders, content experts, and media experts.
- Protocols: In addition to post creation, which largely occurred before launching the intervention, protocols for handling questions and comments, promoting audience engagement, and scheduling posts were useful for delegating tasks and enhancing the reproducibility of dynamic interventions.

Designing a successful web-based intervention required an iterative and responsive approach to the development and testing of post content, delivery processes, and engagement strategies. Our process involved continuous refinement of Facebook posts, ensuring that the posts were relevant, engaging, and theoretically grounded. We built, tested, and revised messages based on feedback from participants, allowing for an agile development process that incorporated 4 groups of experts: a core intervention post development team, health behavior content experts, emerging adult and stakeholder organization advisory boards, and an administrative core. Organizing these expert stakeholders had implications for staffing and resources because it required defining roles, maintaining iterative communication, providing leadership, and delegating. Describing these components of the community-engaged research process expands existing literature by demonstrating realistic timelines, resources, and personnel requirements. Our multi-round, expert-informed development process reflects an agile, person-based approach consistent with recommendations from Yardley et al [50], who emphasize iterative refinement grounded in end user perspectives. Unlike traditional static digital interventions, our approach involved continuous testing and revision across multiple stakeholder groups to increase the ability of participants to enroll in and engage with our content, aligning with emerging literature calling for more engaging, adaptive, and co-designed digital health tools [51]. Our iterative process enabled us to balance the needs and priorities of the communities we aimed to engage—considering factors such as content sources, design elements, and stylistic choices—alongside insights from health behavior content experts and best practices for Facebook engagement. While prior social media–based cancer prevention interventions have largely targeted older adult or urban populations [52,53], our focus on rural EAs (ages 18-26 years) is rare in digital intervention research. This population-specific focus expands the literature by demonstrating how social media messaging must be contextually adapted to audience characteristics, including rural identity and platform use patterns.

This intervention was not only about delivering information but also about motivating audiences to critically engage with web-based content, underscoring the importance of strategic engagement strategies. Given the widespread desire for reliable, expert-driven web-based health content, our strategy involved providing evidence-based information in the digital space. Unlike most interventions that provide one-time educational modules on media literacy [54-57], ours provides media literacy education via real-time sourcing and question-and-answer opportunities, improving responsiveness through timely information correction and availability of content experts to address information gaps. A unique contribution of our project is insight into the digital skillsets required for creating a web-based intervention (eg, web-based platform format, digital editing, algorithm literacy). The intervention was fundamentally community-driven, shaped by ongoing formative research, engagement with stakeholders, and direct interactions with EAs. Recognizing that there is no singular “perfect” message or approach, we focused on key considerations to guide content selection and refinement (eg, source material, visual appeal, representativeness of rurality). While refining our messaging, we also applied a system to assess message acceptability and audience reception through multiple surveys with the target population, whereas most social media-based health messaging studies lack sufficient survey pretesting with small samples [58,59]. This was of utmost importance because it helped us understand which messages were and were not engaging to rural EAs, beyond our EAAB.

Two additional strategies for web-based intervention development emerged. The first is to include information sources that feel reliable to participants, and here we acknowledge that participant perceptions of reliability are critical, particularly given that rural audiences likely differ from urban audiences in their trust patterns in health information sources [60]. A key component of our intervention was training users to assess the quality of web-based information and equipping them with the skills to evaluate content critically, which we refer to as media education. Ideally, this media education will foster the perception of our feed as a credible and dependable resource, as well as help EAs make informed decisions about information from competing sources. Second, including content experts in user-generated engagement videos was seen as an important strategy to enhance the credibility of the research team and the intervention health messages. User-generated engagement videos are a known strategy in advertising and web-based engagement to build trust and authenticity [61], but are scarcely accepted in academic research, which often values professionally designed video content featuring influencers or clinical experts [62].

Designing posts that are theoretically informed and community-engaged required an iterative process of review and revision. In our case, it took two and a half years to develop 400 theoretically informed posts by a team that included 7 media experts, 11 health behavior content experts, 29 community advisors, 26 pilot study participants, and 297 formative survey research participants. The extent of this effort is a likely reason most social media–based interventions fail to incorporate theory [63-66]. To balance structure with responsiveness, we adopted an agile development process, starting with an initial library of posts while continuously refining and expanding content in response to EAAB and SOAB discussions, emerging health information trends, and relevant current events. A yearlong intervention was launched in a randomized trial in March 2025 with twice-daily posts. At the time of the trial launch, nearly half of the posts were developed, with the remaining portion being produced in near real-time to maintain relevance and responsiveness to current events, and reactions and comments from EA users. Our description of the web-based intervention process provides rationale and justification for the inclusion of stakeholder groups, professional networks, and research personnel’s effort to iteratively design a robust web-based intervention.

The pilot test provided valuable insights into the potential acceptability of our messaging and the demographics of our audience. A key focus was examining the prevalence of cancer risk factors and understanding how our messages resonated with a group of EAs by how they engaged with the messages. One of the key lessons learned during the pilot study was the necessity of engaging with audience comments in real-time—a departure from traditional public health communication approaches that historically emerge from larger organizations and government, with little back-and-forth interaction with individuals. This required the development of a structured moderator protocol, including guidelines for managing post schedules and crafting thoughtful reactions to audience engagement, including responding to questions. We relied on this dynamic process to uncover trends in user engagement and tailor our final posts and schedules accordingly. This approach should ensure that our intervention remains relevant, engaging, and impactful.

Interestingly, none of the comments we received during the pilot study actively disputed our messages. Instead, they reflected EAs’ genuine desire for more information, highlighting the growing complexity of the web-based information ecosystem and the challenges EAs face in navigating it. Determining credible information has become increasingly challenging for the public, underscoring the need for clear, accessible, and responsive health communication strategies. Pretesting participants’ views of the credibility of national and local sources was an essential early step in intervention development that should repeated as views of national and local health sources may evolve over time. Adaptability in web-based public health interventions was also essential to ensure that messaging remained both informative and engaging in an ever-evolving digital landscape. For example, health information from a local source may be viewed as credible because of its familiarity to participants or ability to relate it to a local context while information from a national source may be seen as credible because of name recognition and large resources to access key information, demonstrating the nuance of how participants perceive credibility and the need to determine and respond to participants’ perceptions.

### Limitations

Several limitations to the generalizability of this analysis of creating web-based health communication should be acknowledged. Our study targeted EAs, and our analysis may be limited to this age group, which is highly media-savvy and among the heaviest users of digital platforms. Our development process may also apply to interventions for middle-aged and older adults who are also active on the web. However, it may not fully translate as these older groups tend to have different web-based engagement patterns (eg, higher use of different platforms). Additionally, this formative research focused on the Mountain West region, a geographic area with a highly rural demographic composition compared with the rest of the United States, which may additionally limit the generalizability of our findings. Furthermore, the intervention is currently limited to the Facebook platform for its wide use (even among EAs) and private group function that enables us to exercise experimental control in the randomized study design. However, many Americans use other platforms for different media formats and topics. For instance, YouTube and TikTok offer primarily video-based content that may appeal to different demographics or enhance engagement better than Facebook alone can achieve. Future research could explore multi-platform strategies to extend the reach and impact of web-based interventions across EAs and other audiences.

The methods also had 2 limitations. The sample size for the pilot intervention was generally small, as is common in intervention development research [67,68], limiting the representativeness of the EA population and the power of the statistical tests. While the measures used in the pilot study are well-established with good psychometric properties, they rely on self-report, which may produce social desirability biases or recall bias. The larger trial is designed to conduct validation methods of these measures as well. However, we did include observational measures (ie, Facebook metrics) of engagement with the intervention feed in the pilot study.

### Conclusions and Implications

We described a theory-informed community-engaged process for developing a web-based health behavior change intervention, PEAK Wellness. The methods and results provide a novel roadmap for developing community-driven web-based interventions. Community-engaged strategies improve the relevance, feasibility, and cultural fit of health behavior interventions by incorporating community priorities and contextual knowledge into design and pilot-testing. Such approaches can increase recruitment, retention, and implementation potential, while promoting equity and shared ownership, which is especially important in social media interventions that are implemented in geographically diverse areas. These benefits, however, come with added demands for time, partnership building, and iterative adaptation. The resources and effort required to appropriately engage stakeholders, researchers, and the target population, rural EAs, were substantial but essential for developing an engaging and acceptable intervention. This study provides insight that can be used to enhance the rigor and reproducibility of social media intervention designs and facilitate realistic planning of procedures for future web-based health behavior interventions. Testing of the PEAK Wellness intervention has recently commenced in a rigorous pragmatic trial, using a randomized stepped-wedge design, enrolling EAs aged 18-26 years in rural counties in the Western United States. Our dynamic, iterative intervention development process will allow us to remain responsive to the evolving digital landscape while maintaining a foundation of evidence-based communication to promote cancer risk reduction among our sample of rural EAs.

## Appendix

Multimedia Appendix 1 Supplemental tables.

Multimedia Appendix 2 PEAK Wellness Chat Pilot Pretest Survey.

Multimedia Appendix 3 STROBE checklist.

## Abbreviations

EA: emerging adult
EAAB: Emerging Adult Advisory Board
HPV: human papillomavirus
REDCap: Research Electronic Data Capture
RUCC: Rural Urban Continuum Codes
SOAB: Stakeholder Organization Advisory Board
STROBE: Strengthening the Reporting of Observational Studies in Epidemiology

## Data Availability

The datasets generated or analyzed during this study are not publicly available due to participant privacy and confidentiality, but are available from the corresponding author on reasonable request.
