# Supplementary material for: A Web-Based Cancer Prevention Intervention for Rural Emerging Adults: Mixed Methods Development and Pilot-Testing Study
Source: J Med Internet Res. 2026 Jan 8;28:e80803. doi: 10.2196/80803 (PMC12828312; doi:10.2196/80803)
Supplement: Multimedia Appendix 1 [file jmir_v28i1e80803_app1.docx]

| **Table S1. Theoretical and engagement features of posts and examples** | | | | |
| --- | --- | --- | --- | --- |
| **Mediator** | **Definition** | **Behavior Examples** | **Post Messaging Example 1** | **Mediator Definition Sources** |
| Intrinsic Motivation | Motivation to achieve goals in the areas of personal development, community, and meaningful relationships.  Internal sources of motivation such as a need to gain knowledge or independence. A person does something because they find it rewarding/enjoyable, and the outcome satisfies basic psychological needs for autonomy, competence, and relatedness. | A person plays intramural sports or participates in certain exercises (i.e., running, cycling, skiing, etc.) because they enjoy the activity and maybe being with friends.  A person volunteers in a cause they believe in because it gives them internal satisfaction.  A person finds a thrill in competition and so competes in a game that does not have any prizes for winning.  A person decides to quit using tobacco products because they want to be healthier. | Better health starts minutes after your last cigarette or pull on a vape, but it can last a lifetime. Take the first step today. If you need help quitting, we will drop a resource in the comments.  https://www.tiktok.com/@phil.trann/video/7132905853828549930?is_from_webapp=1&sender_device=pc&web_id=7182997283001845294 | Intrinsic Motivation (Deci, 1975)  Self-Determination Theory (Deci 2017) |
| Extrinsic Motivation | Motivation comes from the instrumental value of an activity; it is a means to an end.  Motivation from outside, a person does something because they will get an external reward or gain such as money, fame, power, or avoiding consequences. | The reason for exercising is to achieve a more physically attractive look.  Only having one drink when going out to avoid a hangover the next day or to save money.  Using a QuitLine to help with quitting tobacco use. | Excessive drinking, or binge drinking, can be costly in terms of how it affects your health AND your wallet. The cost of multiple drinks really adds up! Cutting back and moderating drinking can help you save money. We could all use extra cash for everyday expenses right now!  https://www.niaaa.nih.gov/publications/brochures-and-fact-sheets/binge-drinking | Intrinsic Motivation (Deci 1975)  Self-Determination Theory (Deci 2017) |
| Social Support | Social support is positively related to the satisfaction of psychological needs (i.e., relatedness, competence, autonomy), which, in turn, is related positively to perceptions of self-determination and then behavior change intention. Through our relationships and interactions with others, we can foster or thwart well-being and personal growth.  The perception of encouragement and support a person receives from his or her social network. | Starting a new exercise program with friends to keep one another accountable.  Alcoholics Anonymous is a program designed to help alcoholics quit alcohol by providing peer-led mutual aid. These groups help people with behavior change when they are unable to self-regulate. | Poll in Facebook Feed: Sun protection isn't that exciting, but it is important to keep your skin safe. What are your go-to ways to prevent sunburn? I always have sunglasses and a hat with me. Answer with what's easiest for you below!  Response Options: Sunglasses, Hat, Sunscreen, Long-sleeves and/or pants | Intrinsic Motivation (Deci 1975)  Self-Determination (George, 2013)  Glanz, K., Rimer, B. K., & Viswanath, K. (Eds.). (2015). Health behavior: Theory, research, and practice. Chapter 9: HOW INDIVIDUALS, ENVIRONMENTS, AND HEALTH BEHAVIORS INTERACT |
| Relatedness | The need to feel close and connected/ a sense of belonging and attachment to others. | Joining a neighborhood jogging club to become more active versus taking up jogging as a solo activity.  Seeing people you can relate to (i.e., similar age/ethnicity/living situation/behavior patterns, etc.) perform a behavior may increase the desire to also perform the behavior. | Although the highest rate of binge drinking happens among adults between the ages of 18-25 in the US, a recent study finds that some young adults are finding themselves "sober curious". These Gen Zers social lives are centered on coffee shops, restaurants, and new experiences with friends instead of bars and clubs. What is your favorite alcohol-free activity to do with friends?  https://www.bbc.com/worklife/article/20220920-why-gen-zers-are-growing-up-sober-curious | Intrinsic Motivation (Deci 1975)  Self-Determination (George, 2013) |
| Personal Goals | An individual’s desires for their current or future lives. | Quitting smoking to improve one's ability to be active.  Someone diagnosed with pre-diabetes exercises and eats healthier to prevent developing diabetes.  Starting a new training program to achieve a PR in a running or cycling race. | With so much conflicting information about how to eat healthy, making changes to your diet can feel overwhelming. Use these ideas to get started. For more ideas, check out this article: https://anschutzwellness.com/change-ordinary-recipes-extraordinarily-healthy-dishes-2/ | Intrinsic Motivation (Deci 1975)  Personal Goals (Schippers 2020) |
| Ability to Control | People need to feel in control of their own behaviors and goals. This sense of being able to take direct action that will result in real change plays a major part in helping people feel self-determined. | A person who wants to quit smoking finds a way to do so that works for them instead of doing what their father did.  A person chooses to stop drinking for the night after two drinks.  A person chooses not to eat healthier because the idea of "healthy" they have learned about is unattainable (i.e., only organic foods, no or very little processed foods, etc.) | You might not think of knocking back 4 or 5 drinks in an hour or two as binge drinking, but it is! Binge drinking can have lots of harmful health effects and also increases the likelihood of risky behavior. One way to start taking control of binge drinking is by being mindful of your drinking habits and spacing drinks out. #PACE'EMANDSPACE'EM  https://www.youtube.com/watch?v=5jNEVGj6gS0 | Intrinsic Motivation (Deci 1975)  Self-Determination Theory (Deci 2017) |
| Self-efficacy | A person's confidence in his or her ability to perform a behavior that leads to an outcome. Confidence is enhanced through mastery experiences, social modeling, verbal persuasion, and practice under stress-free conditions. | A person who has successfully lost some weight will feel more confident that they can lose more weight.  A person who went to the beach and avoided getting a sunburn will be more confident they can avoid getting a sunburn in the future. | Many of us reach for not-so-healthy foods when tired, frustrated, or stressed - for me, it's the whole bag of potato chips. If you find yourself doing this, try taking a break to stretch, meditate or go for a walk instead. What mood-boosting activities work best for you?  https://www.health.harvard.edu/blog/how-simply-moving-benefits-your-mental-health-201603289350#:~:text=Regular%20exercise%20may%20boost%20mood,cycling%20briefly%20improved%20their%20symptoms. | Social Cognitive Theory (Bandura 1986)  Glanz, K., Rimer, B. K., & Viswanath, K. (Eds.). (2015). Health behavior: Theory, research, and practice. Chapter 9: HOW INDIVIDUALS, ENVIRONMENTS, AND HEALTH BEHAVIORS INTERACT |
| Response efficacy | A person's beliefs as to whether the recommended action step will actually avoid the threat. | A young person will be more likely to get an HPV vaccine if they know it can prevent cancer or how common it is to get HPV. | Human papillomavirus (HPV) is a sexually transmitted infection (STI) that can cause some cancers. Talking about HPV with anyone, even your doctor, can be difficult or awkward. However, 9 in 10 men and 8 in 10 women will get HPV in their lifetime so having those conversations is important. Here are some tips to help. | Social Cognitive Theory (Bandura 1986) |
| Injunctive Norms | The pressure that people feel to conform to a specific norm. | A person feels like they have to have more than 1-2 drinks at a party or club because everyone else is drinking a lot.  Deciding not to get an HPV vaccine because your family and friends are against it. | Did you know that people ages 18-26 can still get the HPV vaccine if they didn't receive it in their pre-teen years? It's a different dosage (3 doses for young adults vs. 2 as a pre-teen), but it works the same. Have you heard any concerns about getting the HPV vaccine from friends or family? Let us know! | Social Cognitive Theory (Bandura 1986)  Perceived norms  https://etd.ohiolink.edu/apexprod/rws_etd/send_file/send?accession=osu1406215621&disposition=inline#:~:text=Perceived%20norms%20consist%20of%20both,Rimal%20%26%20Real%2C%202003). |
| Descriptive Norms | People’s perceptions of how common a certain behavior is. | Seeing a lot of smoking in the media and believing it is something everyone does. | Do you notice how often you see smoking in movies, shows, video games, or online? Seeing images of smoking increases your chances of starting to smoke. This is because they make smoking seem normal and show it as glamorous, rebellious, and edgy. Check out this video that reports how much smoking is now shown on streaming services, where smoking is all over the screen.  https://www.youtube.com/watch?v=joKmtRz3X6g | Social Cognitive Theory (Bandura 1986)  https://etd.ohiolink.edu/apexprod/rws_etd/send_file/send?accession=osu1406215621&disposition=inline#:~:text=Perceived%20norms%20consist%20of%20both,Rimal%20%26%20Real%2C%202003). |
| Cancer Risk Perceptions | The belief or judgment about the likelihood of one’s personal risk of developing cancer, as well as the probability of benefit from interventions or behavior changes. | A person believes they are at a higher risk for HPV, so they get vaccinated.  A person takes steps to avoid getting a sunburn because they believe it will increase their risk of getting skin cancer. | Avoiding a sunburn is the best thing you can do for skin cancer prevention. But sunburns happen. If you do get burned, here are some tips for caring for your skin. In the future, remember that preventing a sunburn is easier than treating it - use sunscreen, stay in the shade, and wear cover-up clothing when outside. | Social Cognitive Theory (Bandura 1986) |
| Response Cost | Response cost is a special case of a punishment procedure that involves taking away desirable possessions, points, tokens, or privileges in planned, incremental steps following the occurrence of an undesirable behavior or failure to meet a specific goal. That is, **behaving inappropriately or in an undesirable manner costs the individual something they already possess or privileges they currently enjoy and expect to have access to in the future.** | Drinking too much at one time (binge drinking) can result in negative outcomes in the immediate future. | Binge drinking, or more than 4 drinks for women and more than 5 drinks for men in about two hours, increases the risk of harm from drinking. It can increase the chance of blackouts, unintentional pregnancy, and STIs due to unsafe sexual behavior, injuries, and car crashes. | Social Cognitive Theory (Bandura 1986)  Response Cost (Rapport 2005) |
| Compatibility with Values | Value compatibility is a measure of how closely an individual’s personal values and the perceived values of an organization or behavior overlap. Values do not have to be identical. In order to be compatible, values only need to be similar enough to support common causes and avoid clashes over issues important to the individual and the organization or behavior.  The degree to which a behavior that reduces cancer risk is perceived as consistent with the existing values of participants. If the behavior is incompatible with their values, norms, or practices, it will not be adopted as easily. | A person trusts medical science, so they get vaccinated for HPV.  A person likes the way they look with a tan, so they don't practice sun safety when outside.  A person chooses not to regularly exercise because they don't want to join a gym and are unsure of how to get exercise in other ways. | Did you know that people ages 18-26 can still get the HPV vaccine if they didn't receive it in their pre-teen years? It's a different dosage (3 doses for young adults vs. 2 as a pre-teen), but it works the same. Have you heard any concerns about getting the HPV vaccine from friends or family? Let us know! | Cazier JA, Gill M. Values in e-business: testing value compatibility and trust production in e-commerce. Paper presented at: 9th Americas Conference on Information Systems; Tampa, FL; August 4-6, 2003.  Diffusion of Innovations (Rogers 2003) |
| Observable Benefits | The extent to which the results or benefits of reducing the cancer risk behavior are visible. Participants should be able to clearly see the benefit of reducing cancer risk behaviors. I.e., role modeling | A person makes a TikTok video of how their life has improved since they quit vaping/smoking/drinking, | Better health starts minutes after your last cigarette or pull on a vape, but it can last a lifetime. Take the first step today. If you need help quitting, we will drop a resource in the comments.  https://www.tiktok.com/@phil.trann/video/7132905853828549930?is_from_webapp=1&sender_device=pc&web_id=7182997283001845294 | Diffusion of Innovations (Rogers 2003) |
| Simplicity | Ease with which a participant can understand and perform the behavior that reduces cancer risk or stop performing the behavior that increases cancer risk and understand why stopping the behavior reduces cancer risk. | Encouraging someone to make small changes to the way they eat to improve their overall health. | With so much conflicting information about how to eat healthy, making changes to your diet can feel overwhelming. Use these ideas to get started. For more ideas, check out this article:  https://anschutzwellness.com/change-ordinary-recipes-extraordinarily-healthy-dishes-2/ | Diffusion of Innovations (Rogers 2003) |

**Table S2.** Feedback on the Facebook group

| **Characteristic** | **N = 23***^2^* |
| --- | --- |
| I think that I would like to use the #4Corners4Health^1^ Facebook group. |  |
| Strongly disagree | 0 (0%) |
| Disagree | 1 (4.3%) |
| Neither agree nor disagree | 3 (13%) |
| Agree | 14 (61%) |
| Strongly agree | 5 (22%) |
| I think I will find the #4Corners4Health^1^ Facebook group unnecessarily complex. |  |
| Strongly disagree | 6 (26%) |
| Disagree | 10 (43%) |
| Neither agree nor disagree | 6 (26%) |
| Agree | 1 (4.3%) |
| Strongly agree | 0 (0%) |
| I think the #4Corners4Health^1^ Facebook group will be easy to use. |  |
| Strongly disagree | 0 (0%) |
| Disagree | 0 (0%) |
| Neither agree nor disagree | 2 (8.7%) |
| Agree | 11 (48%) |
| Strongly agree | 10 (43%) |
| I think that I would need the support of a technical person to be able to use the #4Corners4Health^1^ Facebook group. |  |
| Strongly disagree | 15 (65%) |
| Disagree | 5 (22%) |
| Neither agree nor disagree | 1 (4.3%) |
| Agree | 2 (8.7%) |
| Strongly agree | 0 (0%) |
| I think that I will find the various functions in the #4Corners4Health^1^ Facebook group were well integrated. |  |
| Strongly disagree | 0 (0%) |
| Disagree | 0 (0%) |
| Neither agree nor disagree | 3 (13%) |
| Agree | 11 (48%) |
| Strongly agree | 9 (39%) |
| I think there will be too much inconsistency in the #4Corners4Health^1^ Facebook group. |  |
| Strongly disagree | 10 (43%) |
| Disagree | 7 (30%) |
| Neither agree nor disagree | 3 (13%) |
| Agree | 3 (13%) |
| Strongly agree | 0 (0%) |
| I imagine that most people would learn to use the #4Corners4Health^1^ Facebook group very quickly. |  |
| Strongly disagree | 0 (0%) |
| Disagree | 0 (0%) |
| Neither agree nor disagree | 2 (8.7%) |
| Agree | 7 (30%) |
| Strongly agree | 14 (61%) |
| I think that I will find the #4Corners4Health^1^ Facebook group very awkward to use. |  |
| Strongly disagree | 8 (35%) |
| Disagree | 8 (35%) |
| Neither agree nor disagree | 3 (13%) |
| Agree | 3 (13%) |
| Strongly agree | 1 (4.3%) |
| I will feel very confident using the #4Corners4Health^1^ Facebook group. |  |
| Strongly disagree | 0 (0%) |
| Disagree | 0 (0%) |
| Neither agree nor disagree | 4 (17%) |
| Agree | 7 (30%) |
| Strongly agree | 12 (52%) |
| I will need to learn a lot of things before I could get going with the #4Corners4Health^1^ Facebook group. |  |
| Strongly disagree | 6 (26%) |
| Disagree | 8 (35%) |
| Neither agree nor disagree | 3 (13%) |
| Agree | 2 (8.7%) |
| Strongly agree | 4 (17%) |
| Overall, I think that I would rate the user-friendliness of the #4Corners4Health^1^ Facebook group as. |  |
| 1 = Worst imaginable | 0 (0%) |
| 2 = Awful | 0 (0%) |
| 3 = Poor | 0 (0%) |
| 4 = OK | 2 (8.7%) |
| 5 = Good | 2 (8.7%) |
| 6 = Excellent | 14 (61%) |
| 7 = Best imaginable | 5 (22%) |
| I did not receive enough information that interests me in the #4Corners4Health^1^ Facebook group. |  |
| Strongly disagree | 6 (26%) |
| Disagree | 11 (48%) |
| Neither agree nor disagree | 2 (8.7%) |
| Agree | 4 (17%) |
| Strongly agree | 0 (0%) |
| I did not feel comfortable posting comments or information in the #4Corners4Health^1^ Facebook group. |  |
| Strongly disagree | 4 (17%) |
| Disagree | 11 (48%) |
| Neither agree nor disagree | 2 (8.7%) |
| Agree | 4 (17%) |
| Strongly agree | 2 (8.7%) |
| I would like to continue to receive the posts and information from the #4Corners4Health^1^ Facebook group. |  |
| Strongly disagree | 1 (4.3%) |
| Disagree | 1 (4.3%) |
| Neither agree nor disagree | 4 (17%) |
| Agree | 12 (52%) |
| Strongly agree | 5 (22%) |
| The posts in the #4Corners4Health^1^ Facebook group were useful to me. |  |
| Strongly disagree | 1 (4.3%) |
| Disagree | 1 (4.3%) |
| Neither agree nor disagree | 3 (13%) |
| Agree | 10 (43%) |
| Strongly agree | 8 (35%) |
| Viewing other users' comments and replies to posts in the #4Corners4Health^1^ Facebook group were useful to me. |  |
| Strongly disagree | 0 (0%) |
| Disagree | 1 (4.3%) |
| Neither agree nor disagree | 3 (13%) |
| Agree | 12 (52%) |
| Strongly agree | 7 (30%) |
| How likely is it that you would use the #4Corners4Health^1^ Facebook group in the future? |  |
| Not likely at all | 1 (4.3%) |
| Somewhat unlikely | 1 (4.3%) |
| Neither likely nor unlikely | 5 (22%) |
| Somewhat likely | 6 (26%) |
| Very likely | 10 (43%) |
| ^1^The #4Corners4Health was later renamed PEAK Wellness Chat  ^2^ n (%) | |
