# Supplementary material for: A Web-Based Cancer Prevention Intervention for Rural Emerging Adults: Mixed Methods Development and Pilot-Testing Study
Source: J Med Internet Res. 2026 Jan 8;28:e80803. doi: 10.2196/80803 (PMC12828312; doi:10.2196/80803)
Supplement: Multimedia Appendix 2 [file jmir_v28i1e80803_app2.pdf]

4Corners Survey

You are being asked to complete this survey to measure six behaviors that can impact the chances of developing cancer later in life.

We are recruiting 25 participants from states in the 4 Corners region of the United States (Arizona, Colorado, New Mexico, Utah) to join a facebook group to pilot test a social media program to help young adults, like you, stay healthy. Your responses to the questions will be kept confidential. Your decision to complete this survey is voluntary, and you may leave the survey at any time. You also may skip any survey questions you do not wish to answer. You can also press the "reset" button if you want to change an answer.

By clicking "next" to begin the survey, you are giving us permission to use your responses.

Total Survey Time (according to REDCap) ________________________________
(mm:ss format)

Survey Date ________________________________

Time 1 ________________________________

First, we want to know how often you use different types of social media, if at all.

About how often, if at all, do you use Instagram?
- ◯ Several times a day
- ◯ About once a day
- ◯ A few times a week
- ◯ Every few weeks
- ◯ Less often
- ◯ Never- I do not use it

About how often, if at all, do you use YouTube?
- ◯ Several times a day
- ◯ About once a day
- ◯ A few times a week
- ◯ Every few weeks
- ◯ Less often
- ◯ Never- I do not use it

About how often, if at all, do you use TikTok?
- ◯ Several times a day
- ◯ About once a day
- ◯ A few times a week
- ◯ Every few weeks
- ◯ Less often
- ◯ Never- I do not use it

Thinking just of Facebook... Do you belong to any Facebook private groups? By private groups, we mean a place where you connect with other Facebook users who are interested in a topic that interests you and membership was approved by a group administrator.
- ◯ Yes
- ◯ No

About how often do you read posts from one of these Facebook private groups?
- ◯ Several times a day
- ◯ About once a day
- ◯ A few times a week
- ◯ Every few weeks
- ◯ Less often

The next questions are about stories you may have heard in the media, including in newspapers, on television, online, in social media, or elsewhere.

Please indicate if you have heard a story about each of the following topics.

	Yes	No
Using nicotine products such as smoking, vaping, and smokeless tobacco	◯	◯
Drinking alcohol	◯	◯
Eating a healthy diet	◯	◯
Being physically active	◯	◯
Getting vaccinated for human papillomavirus (HPV)	◯	◯
Avoiding sunburns	◯	◯

How strongly do you agree or disagree with the following statements about how you use the internet and social media?

I know what health resources are available on the Internet.

◯ Strongly disagree
◯ Disagree
◯ Neither agree nor disagree
◯ Agree
◯ Strongly agree

I have the skills I need to evaluate the health information I find on the Internet.

◯ Strongly disagree
◯ Disagree
◯ Neither agree nor disagree
◯ Agree
◯ Strongly agree

I can tell high quality from low quality health information I find on the Internet.

◯ Strongly disagree
◯ Disagree
◯ Neither agree nor disagree
◯ Agree
◯ Strongly agree

I feel confident using information from the Internet to make health decisions.

◯ Strongly disagree
◯ Disagree
◯ Neither agree nor disagree
◯ Agree
◯ Strongly agree

I know how to verify whether what is shared on social media is correct.

◯ Strongly disagree
◯ Disagree
◯ Neither agree nor disagree
◯ Agree
◯ Strongly agree

I can tell whether information on social media is true or false.

◯ Strongly disagree
◯ Disagree
◯ Neither agree nor disagree
◯ Agree
◯ Strongly agree

Social media sites such as Facebook control what I see on social media.	◯ Strongly disagree<br>◯ Disagree<br>◯ Neither agree nor disagree<br>◯ Agree<br>◯ Strongly agree
Information that I post on social media is permanent.	◯ Strongly disagree<br>◯ Disagree<br>◯ Neither agree nor disagree<br>◯ Agree<br>◯ Strongly agree
The advertisements I see on social media are specifically targeted to my preferences.	◯ Strongly disagree<br>◯ Disagree<br>◯ Neither agree nor disagree<br>◯ Agree<br>◯ Strongly agree
Date 30 days ago	________________________
Date 3 months ago	________________________

Now, we will ask about your health.

Time 2 __________________________________

Social Media Time Elapsed __________________________________

Physical Activity

Think about the time you spend doing different types of physical activity in a typical week. Please answer these questions even if you do not consider yourself to be a physically active person.

Activity at Work

Think first about the time you spend doing work, paid or unpaid, including housework.

Does your work involve moderate-intensity activity that causes small increases in breathing or heart rate such as brisk walking [or carrying light loads] OR vigorous-intensity activity that causes large increases in breathing or heart rate [like carrying or lifting heavy loads, digging or construction work] for at least 10 minutes continuously?

◯ Yes
◯ No

Moderate-Intensity Work Examples

Vigorous-Intensity Work Examples

In a typical week, on how many days do you do moderate or vigorous-intensity activities as part of your work?

◯ 0
◯ 1
◯ 2
◯ 3
◯ 4
◯ 5
◯ 6
◯ 7
(Number of days)

How much time do you spend doing moderate or vigorous-intensity activities at work on a typical day? ______ ______
Hours Minutes

Travel To and From Places

Excluding activities you do for work, do you walk, skateboard or use a bicycle for at least 10 minutes continuously to get to and from places?

◯ Yes
◯ No

In a typical week, on how many days do you walk, skateboard or use a bicycle for at least 10 minutes continuously to get to and from places?

◯ 0
◯ 1
◯ 2
◯ 3
◯ 4
◯ 5
◯ 6
◯ 7
(Number of days)

How much time do you spend walking, skateboarding, or bicycling for travel on a typical day? ______ ______
Hours Minutes

Recreational Activities

Excluding activities you do for work or travel, do you do any moderate-intensity sports, fitness or recreational activities that cause a small increase in breathing or heart rate [such as brisk walking, cycling, or dancing] OR vigorous-intensity sports, fitness or recreational activities that cause large increases in breathing or heart rate [like running or rowing] for at least 10 minutes continuously?

◯ Yes
◯ No

Moderate-Intensity Recreational Activity Examples

Vigorous-Intensity Recreational Activity Examples

In a typical week, on how many days do you do moderate or vigorous-intensity sports, fitness or recreational activities?

◯ 0
◯ 1
◯ 2
◯ 3
◯ 4
◯ 5
◯ 6
◯ 7
(Number of days)

How much time do you spend doing moderate or vigorous-intensity sports, fitness or recreational activities on a typical day? ______ ______
Hours Minutes

Total Time In MVPA (hours)

____

You reported [mvpa_total] hours spent in moderate to vigorous physical activity (work, recreational, and travel combined) on a typical day. Is that correct?

◯ Yes
◯ No

Please revise your responses for time spent in moderate or vigorous activity for work, travel to and from places and recreation.

Tobacco/Nicotine Use

Question	Response
Time 3	______________________
Physical Activity Time Elapsed	______________________
Have you ever used any tobacco or nicotine products (cigarettes, vaping/e-cigarettes, smokeless tobacco, etc.), even one time?	◯ Yes, I currently use tobacco or nicotine products<br>◯ Yes, I experimented with tobacco or nicotine products in the last 30 days<br>◯ Yes, I experimented but have not used tobacco or nicotine products in the last 30 days<br>◯ No, I have never used any tobacco or nicotine products
Have you used any tobacco or nicotine products in the last 30 days?	◯ Yes - Every day<br>◯ Yes - Some days<br>◯ No - Not at all
Have you used any tobacco or nicotine products in the last 7 days?	◯ Yes - Every day<br>◯ Yes - Some days<br>◯ No - Not at all
On a scale of 1 to 10 (where 1 is no thought of quitting and 10 is taking action to quit), how likely are you to quit tobacco/nicotine in the next month?	◯ 1 (No thought of quitting)<br>◯ 2<br>◯ 3 (Think I need to consider quitting someday)<br>◯ 4<br>◯ 5 (Think I should quit but not quite ready)<br>◯ 6<br>◯ 7 (Starting to think about quitting)<br>◯ 8<br>◯ 9<br>◯ 10 (Taking action to quit)
What type of tobacco or nicotine products have you ever used? (Select all that apply)	☐ Vaping/e-cigarettes<br>☐ Cigarettes<br>☐ Smokeless tobacco (chew, dip, snus, etc.)<br>☐ Cigars<br>☐ Other oral nicotine product (velo, lucy, rogue, etc.)<br>☐ Nicotine patches, nicotine gum, nicotine lozenges<br>☐ None

Sun Exposure/Sun Protection

Time 4

____

Tobacco Time Elapsed

____

Please think carefully about what you did each day in the past 3 months ([date_3_mo_ago] to [survey_date]) between 10 am and 4 pm.

How many days were you outdoors in the past 3 months between 10 am and 4 pm?

- ◯ 0
- ◯ 1
- ◯ 2
- ◯ 3
- ◯ 4
- ◯ 5
- ◯ 6
- ◯ 7
- ◯ 8
- ◯ 9
- ◯ 10
- ◯ 11
- ◯ 12
- ◯ 13
- ◯ 14
- ◯ 15
- ◯ 16
- ◯ 17
- ◯ 18
- ◯ 19
- ◯ 20
- ◯ 21
- ◯ 22
- ◯ 23
- ◯ 24
- ◯ 25
- ◯ 26
- ◯ 27
- ◯ 28
- ◯ 29
- ◯ 30
- ◯ 31
- ◯ 32
- ◯ 33
- ◯ 34
- ◯ 35
- ◯ 36
- ◯ 37
- ◯ 38
- ◯ 39
- ◯ 40
- ◯ 41
- ◯ 42
- ◯ 43
- ◯ 44
- ◯ 45
- ◯ 46
- ◯ 47
- ◯ 48
- ◯ 49
- ◯ 50
- ◯ 51
- ◯ 52
- ◯ 53
- ◯ 54
- ◯ 55
- ◯ 56
- ◯ 57
- ◯ 58
- ◯ 59
- ◯ 60
- ◯ 61
- ◯ 62
- ◯ 63
- ◯ 64
- ◯ 65
- ◯ 66
- ◯ 67
- ◯ 68

	◯ 69<br>◯ 70<br>◯ 71<br>◯ 72<br>◯ 73<br>◯ 74<br>◯ 75<br>◯ 76<br>◯ 77<br>◯ 78<br>◯ 79<br>◯ 80<br>◯ 81<br>◯ 82<br>◯ 83<br>◯ 84<br>◯ 85<br>◯ 86<br>◯ 87<br>◯ 88<br>◯ 89<br>◯ 90<br>(days)
How many times, if any, has your skin been sunburned in the past 3 months with redness or pain that lasted more than 24 hours?	◯ 0<br>◯ 1<br>◯ 2<br>◯ 3<br>◯ 4,<br>◯ 5<br>◯ 6<br>◯ 7<br>◯ 8<br>◯ 9<br>◯ 10 or more<br>(Number of sunburns)
How often do you do the following behaviors to protect your skin from the sun when outdoors between 10 am and 4 pm, on a warm and sunny day?	
Apply sunscreen with an SPF 15 or greater on my face by using aftershave, face lotion or make-up.	◯ Never<br>◯ Rarely<br>◯ Sometimes<br>◯ Often<br>◯ Always
Apply sunscreen with an SPF 15 or greater on my exposed body parts (not including aftershave, face lotion or make-up).	◯ Never<br>◯ Rarely<br>◯ Sometimes<br>◯ Often<br>◯ Always
Wear long-sleeved shirts and long pants.	◯ Never<br>◯ Rarely<br>◯ Sometimes<br>◯ Often<br>◯ Always
Wear a hat with a wide-brim all the way around. (This includes a hat with a flap in the back that protects the ears and the neck)	◯ Never<br>◯ Rarely<br>◯ Sometimes<br>◯ Often<br>◯ Always

Limit my time in the sun.	◯ Never<br>◯ Rarely<br>◯ Sometimes<br>◯ Often<br>◯ Always
Stay mostly in the shade.	◯ Never<br>◯ Rarely<br>◯ Sometimes<br>◯ Often<br>◯ Always
What is your eye color?	◯ Grey<br>◯ Green<br>◯ Blue<br>◯ Hazel<br>◯ Brown
What is your natural hair color without artificial hair dye?	◯ Red<br>◯ Blonde<br>◯ Light brown<br>◯ Dark brown<br>◯ Black
What would happen to your skin if it was repeatedly exposed to bright sunlight in the summer without any protection. Would it:	◯ Get no suntan at all or only freckled<br>◯ Get mildly or occasionally tanned<br>◯ Get moderately tanned<br>◯ Get very brown or deeply tanned

During the past 3 months ([date_3_mo_ago] to [survey_date]), how many total days did you expose your skin to ultraviolet light in order to get a tan, that is sunbathe? Include all days when you exposed your skin to the sun or to indoor tanning lights such as in a tanning bed to get a tan.

◯ 0
◯ 1
◯ 2
◯ 3
◯ 4
◯ 5
◯ 6
◯ 7
◯ 8
◯ 9
◯ 10
◯ 11
◯ 12
◯ 13
◯ 14
◯ 15
◯ 16
◯ 17
◯ 18
◯ 19
◯ 20
◯ 21
◯ 22
◯ 23
◯ 24
◯ 25
◯ 26
◯ 27
◯ 28
◯ 29
◯ 30
◯ 31
◯ 32
◯ 33
◯ 34
◯ 35
◯ 36
◯ 37
◯ 38
◯ 39
◯ 40
◯ 41
◯ 42
◯ 43
◯ 44
◯ 45
◯ 46
◯ 47
◯ 48
◯ 49
◯ 50
◯ 51
◯ 52
◯ 53
◯ 54
◯ 55
◯ 56
◯ 57
◯ 58
◯ 59
◯ 60
◯ 61
◯ 62
◯ 63
◯ 64
◯ 65
◯ 66
◯ 67
◯ 68

	◯ 69<br>◯ 70<br>◯ 71<br>◯ 72<br>◯ 73<br>◯ 74<br>◯ 75<br>◯ 76<br>◯ 77<br>◯ 78<br>◯ 79<br>◯ 80<br>◯ 81<br>◯ 82<br>◯ 83<br>◯ 84<br>◯ 85<br>◯ 86<br>◯ 87<br>◯ 88<br>◯ 89<br>◯ 90<br>(Days)
Sunbathing DK	◯ Don't know<br>◯ I prefer not to answer

Human Papillomavirus Prevention

Time 5

____

Sun Safety Time Elapsed

____

HPV is the Human Papillomavirus.
Have you ever received an HPV vaccine?

- ◯ Yes
- ◯ No
- ◯ Don't know

How old were you when you received your first HPV vaccine?

- ◯ 17 years or younger
- ◯ 18 years or older
- ◯ Don't know

How many doses of the HPV vaccine have you received?

- ◯ 1
- ◯ 2
- ◯ 3
- ◯ Don't know

Did you complete all recommended doses of the HPV vaccine?

- ◯ Yes
- ◯ No
- ◯ Don't know

Alcohol Use

Time 6

____

HPV Time Elapsed

____

Binge drinking definition by sex

____

How often do you have a drink containing alcohol?

◯ Never
◯ Monthly or less
◯ 2-4 times a month
◯ 2-3 times a week
◯ 4 or more times a week

Please review the graphic below depicting a "standard alcoholic drink" for various beverages when reporting how many standard drinks you consume. Please keep in mind that a larger container than is shown in this graphic may include more than one standard drink.

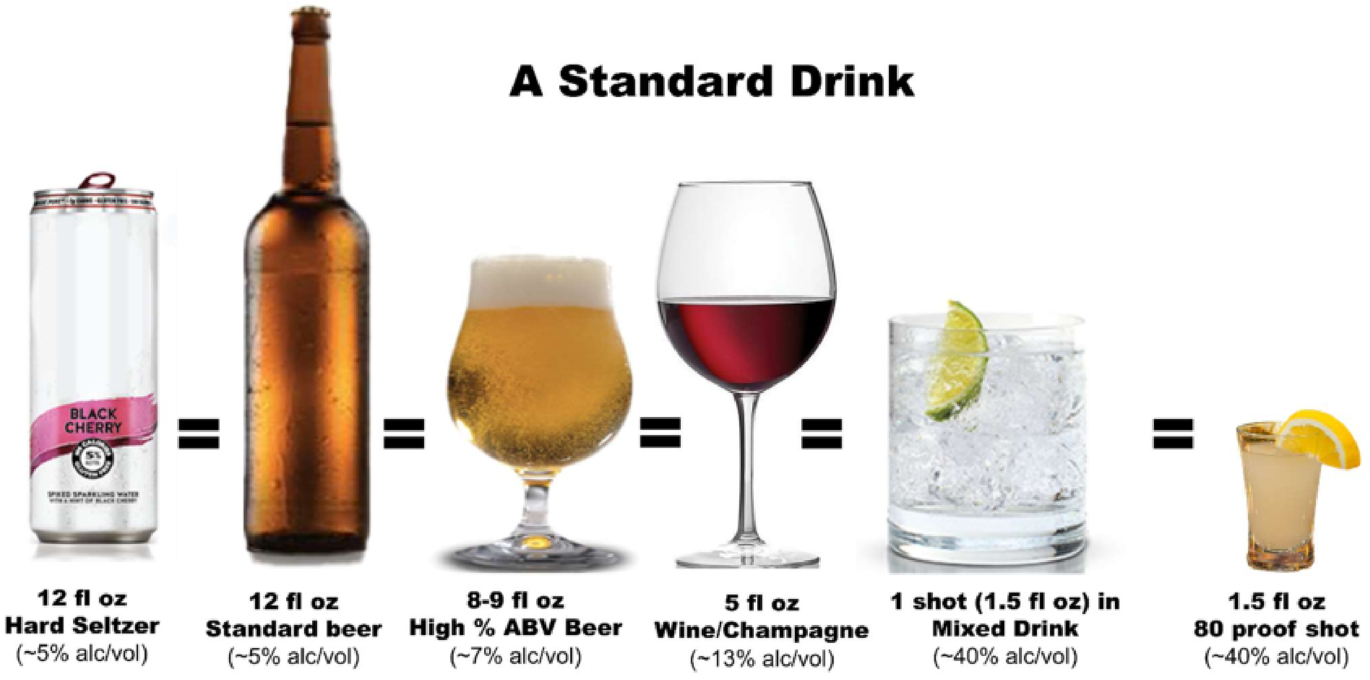

How many standard drinks containing alcohol do you have on a typical day?

◯ 1 or 2
◯ 3 to 4
◯ 5 to 6
◯ 7 to 9
◯ 10 or more

How often do you have six or more drinks on one occasion?

◯ Never
◯ Less than monthly
◯ Monthly
◯ Weekly
◯ Daily or almost daily

During the past 30 days (since [date_30_d_ago]), on how many days did you have [binge_drink_calc] or more drinks on the same occasion? By "occasion," we mean at the same time or within a couple of hours of each other?

◯ 0
◯ 1
◯ 2
◯ 3
◯ 4
◯ 5
◯ 6
◯ 7
◯ 8
◯ 9
◯ 10
◯ 11
◯ 12
◯ 13
◯ 14
◯ 15
◯ 16
◯ 17
◯ 18
◯ 19
◯ 20
◯ 21
◯ 22
◯ 23
◯ 24
◯ 25
◯ 26
◯ 27
◯ 28
◯ 29
◯ 30
(Days)

Eating Behaviors

Time 7

____

Alcohol Time Elapsed

____

How strongly do you agree with the following statements?

Many of my friends and family care about eating healthy food.

- ○ Strongly disagree
- ○ Disagree
- ○ Neither agree nor disagree
- ○ Agree
- ○ Strongly agree

I usually eat dinner with other people.

- ○ Strongly disagree
- ○ Disagree
- ○ Neither agree nor disagree
- ○ Agree
- ○ Strongly agree

Most unhealthy foods taste better than healthy foods

- ○ Strongly disagree
- ○ Disagree
- ○ Neither agree nor disagree
- ○ Agree
- ○ Strongly agree

I tend to eat on the run

- ○ Strongly disagree
- ○ Disagree
- ○ Neither agree nor disagree
- ○ Agree
- ○ Strongly agree

The types of food I eat affect my health

- ○ Strongly disagree
- ○ Disagree
- ○ Neither agree nor disagree
- ○ Agree
- ○ Strongly agree

People my age don't need to be concerned about their eating habits

- ○ Strongly disagree
- ○ Disagree
- ○ Neither agree nor disagree
- ○ Agree
- ○ Strongly agree

During the past 30 days, about how often have you. . .

Bought fresh vegetables at the grocery store

- ○ Never
- ○ Once a month
- ○ Once a week
- ○ Twice a week
- ○ 3 or more times a week
- ○ Daily

Prepared an entire dinner for 2 or more people	◯ Never<br>◯ Once a month<br>◯ Once a week<br>◯ Twice a week<br>◯ 3 or more times a week<br>◯ Daily

We would like to get a sense of how you make decisions. We know that your preferences can impact your decisions.

To show that you've read this question, just go ahead and select orange and blue among the responses below, no matter what your favorite color is. Just ignore the question below and select orange and blue.<br><br>What is your favorite color?	☐ Red<br>☐ Orange<br>☐ Yellow<br>☐ Green<br>☐ Blue<br>☐ Purple<br>☐ Don't know

Diet

Time 8

____

Eating Behavior Time Elapsed

____

Date

____

This Month (enter as full name of month i.e., April)

____

The next questions ask you about what you ate in the past month (30 days). We will ask about several types of food and how often you eat them (monthly, weekly, or daily). Please think about each food as you answer the questions.

During the past month, how often did you eat hot or cold cereals?

Choose only one.

- ◯ Never
- ◯ 1 time in last month
- ◯ 2-3 times in last month
- ◯ 1 time per week
- ◯ 2 times per week
- ◯ 3-4 times per week
- ◯ 5-6 times per week
- ◯ 1 time per day
- ◯ 2 or more times per day

During the past month, what kind of cereal did you usually eat?

- ◯ High Fiber Cereal (e.g., All Bran, Kashi Go Lean)
- ◯ Sweetened Cereal (e.g., Frosted Flakes, Cocoa Puffs, Frosted Mini Wheats, Captain Crunch)
- ◯ Unsweetened Whole Grain Cereal (Cheerios, Life Original)
- ◯ Unsweetened Non-Whole Grain Cereal (Rice Krispies, Corn Flakes, Special K)
- ◯ Oatmeal
- ◯ Granola
- ◯ Other, please describe:

Other Cereal, please specify:

____

Cereal #1 Food Code

____

If there was another type of cereal that you usually ate during the past month, what kind was it?

- ◯ None
- ◯ High Fiber Cereal (e.g., All Bran, Kashi Go Lean)
- ◯ Sweetened Cereal (e.g., Frosted Flakes, Cocoa Puffs, Frosted Mini-Wheat, Captain Crunch)
- ◯ Unsweetened Whole Grain Cereal (Cheerios, Life Original)
- ◯ Unsweetened Non-Whole Grain Cereal (Rice Krispies, Corn Flakes, Special K)
- ◯ Oatmeal
- ◯ Granola
- ◯ Other, please describe:

Other Cereal, please specify:

____

Cereal #2 Food Code

____

During the past month, how often did you have any milk (either to drink or in cereal)?

Include regular milks, chocolate or flavored milks, lactose-free milk, or buttermilk.

Do NOT include alternative milks (soy, almond or oat milk, etc.) or small amounts of milk in coffee or tea

- ◯ Never
- ◯ 1 time in last month
- ◯ 2-3 times in last month
- ◯ 1 time per week
- ◯ 2 times per week
- ◯ 3-4 times per week
- ◯ 5-6 times per week
- ◯ 1 time per day
- ◯ 2 or more times per day

During the past month, what kind of milk did you usually drink?

- ◯ Whole or regular milk
- ◯ 2% fat or reduced-fat milk
- ◯ 1%, 1/2%, or low-fat milk
- ◯ Fat-free, skim or nonfat milk
- ◯ Soy milk
- ◯ Alternative milk

If other, what kind of milk do you drink?

____

During the past month, how often did you drink regular soda or pop that contains sugar?

Do NOT include diet soda.

- ◯ Never
- ◯ 1 time in last month
- ◯ 2-3 times in last month
- ◯ 1 time per week
- ◯ 2 times per week
- ◯ 3-4 times per week
- ◯ 5-6 times per week
- ◯ 1 time per day
- ◯ 2 or more times per day
- ◯ 4-5 times per day
- ◯ 6 or more times per day

During the past month, how often did you drink 100% pure fruit juices such as orange, mango, apple, grape, or pineapple juices?

Do NOT include fruit-flavored drinks with added sugar or fruits juice you made at home and added sugar to.

- ◯ Never
- ◯ 1 time in last month
- ◯ 2-3 times in last month
- ◯ 1 time per week
- ◯ 2 times per week
- ◯ 3-4 times per week
- ◯ 5-6 times per week
- ◯ 1 time per day
- ◯ 2 or more times per day
- ◯ 4-5 times per day
- ◯ 6 or more times per day

During the past month, how often did you drink coffee or tea that had sugar or honey added to it?

Include coffee and tea you sweetened yourself as well as presweetened tea and coffee drinks such as Arizona Iced Tea and Frappuccinos.

Do NOT include artificially sweetened coffee or diet tea.

- ◯ Never
- ◯ 1 time in last month
- ◯ 2-3 times in last month
- ◯ 1 time per week
- ◯ 2 times per week
- ◯ 3-4 times per week
- ◯ 5-6 times per week
- ◯ 1 time per day
- ◯ 2 or more times per day
- ◯ 4-5 times per day
- ◯ 6 or more times per day

During the past month, how often did you drink sweetened fruit drinks, sports or energy drinks, such as Kool-Aid, lemonade, Hi-C, cranberry drinks, Gatorade, Red Bull, or Vitamin Water?<br><br>Include fruit juices you made at home and added sugar to.<br><br>Do NOT include diet drinks or artificially sweetened drinks.	◯ Never<br>◯ 1 time in last month<br>◯ 2-3 times in last month<br>◯ 1 time per week<br>◯ 2 times per week<br>◯ 3-4 times per week<br>◯ 5-6 times per week<br>◯ 1 time per day<br>◯ 2 or more times per day<br>◯ 4-5 times per day<br>◯ 6 or more times per day
During the past month, how often did you eat fruit?<br><br>Include fresh, frozen, or canned fruit.<br><br>Do NOT include juices.	◯ Never<br>◯ 1 time in last month<br>◯ 2-3 times in last month<br>◯ 1 time per week<br>◯ 2 times per week<br>◯ 3-4 times per week<br>◯ 5-6 times per week<br>◯ 1 time per day<br>◯ 2 or more times per day
During the past month, how often did you eat green leafy or lettuce salad, with or without other vegetables?	◯ Never<br>◯ 1 time in last month<br>◯ 2-3 times in last month<br>◯ 1 time per week<br>◯ 2 times per week<br>◯ 3-4 times per week<br>◯ 5-6 times per week<br>◯ 1 time per day<br>◯ 2 or more times per day
During the past month, how often did you eat any kind of fried potatoes, including french fries, home fries, or hash brown potatoes?	◯ Never<br>◯ 1 time in last month<br>◯ 2-3 times in last month<br>◯ 1 time per week<br>◯ 2 times per week<br>◯ 3-4 times per week<br>◯ 5-6 times per week<br>◯ 1 time per day<br>◯ 2 or more times per day
During the past month, how often did you eat any other kind of potatoes, such as baked, boiled, mashed potatoes, sweet potatoes, or potato salad?	◯ Never<br>◯ 1 time in last month<br>◯ 2-3 times in last month<br>◯ 1 time per week<br>◯ 2 times per week<br>◯ 3-4 times per week<br>◯ 5-6 times per week<br>◯ 1 time per day<br>◯ 2 or more times per day
During the past month, how often did you eat refried beans, baked beans, beans in soup, pork and beans, or any other type of cooked dried beans?<br><br>Do NOT include green beans.	◯ Never<br>◯ 1 time in last month<br>◯ 2-3 times in last month<br>◯ 1 time per week<br>◯ 2 times per week<br>◯ 3-4 times per week<br>◯ 5-6 times per week<br>◯ 1 time per day<br>◯ 2 or more times per day

During the past month, not including what you just told me about (green salads, potatoes, cooked or dried beans), how often did you eat other vegetables?	◯ Never<br>◯ 1 time in last month<br>◯ 2-3 times in last month<br>◯ 1 time per week<br>◯ 2 times per week<br>◯ 3-4 times per week<br>◯ 5-6 times per week<br>◯ 1 time per day<br>◯ 2 or more times per day
During the past month, how often did you eat pizza?<br><br>Include frozen pizza, fast food pizza, and homemade pizza.	◯ Never<br>◯ 1 time in last month<br>◯ 2-3 times in last month<br>◯ 1 time per week<br>◯ 2 times per week<br>◯ 3-4 times per week<br>◯ 5-6 times per week<br>◯ 1 time per day<br>◯ 2 or more times per day
During the past month, how often did you have Mexican-type salsa made with tomato?	◯ Never<br>◯ 1 time in last month<br>◯ 2-3 times in last month<br>◯ 1 time per week<br>◯ 2 times per week<br>◯ 3-4 times per week<br>◯ 5-6 times per week<br>◯ 1 time per day<br>◯ 2 or more times per day
During the past month, how often did you have tomato sauces such as with spaghetti or noodles or mixed into foods such as lasagna?<br><br>Do NOT include tomato sauce on pizza.	◯ Never<br>◯ 1 time in last month<br>◯ 2-3 times in last month<br>◯ 1 time per week<br>◯ 2 times per week<br>◯ 3-4 times per week<br>◯ 5-6 times per week<br>◯ 1 time per day<br>◯ 2 or more times per day
During the past month, how often did you eat red meat, such as beef, pork, ham, or hamburger?<br><br>Include red meat you had in sandwiches, lasagna, stew, and other mixtures. Red meats also include veal and lamb.<br><br>Do NOT include chicken, turkey, or seafood.	◯ Never<br>◯ 1 time in last month<br>◯ 2-3 times in last month<br>◯ 1 time per week<br>◯ 2 times per week<br>◯ 3-4 times per week<br>◯ 5-6 times per week<br>◯ 1 time per day<br>◯ 2 or more times per day
During the past month, how often did you eat any processed meat such as bacon, lunch meats, sausage or hot dogs?<br>*Processed meats are those preserved by smoking, curing, or salting or by the addition of preservatives.<br><br>Include processed meats you had in sandwiches, soups, pizza, casseroles, and other mixtures. Ex: pastrami, salami, sausages, bratwursts, bologna and spam	◯ Never<br>◯ 1 time in last month<br>◯ 2-3 times in last month<br>◯ 1 time per week<br>◯ 2 times per week<br>◯ 3-4 times per week<br>◯ 5-6 times per week<br>◯ 1 time per day<br>◯ 2 or more times per day

During the past month, how often did you eat any kind of cheese?

Include cheese as a snack, cheese on burgers, sandwiches, and cheese in foods such as lasagna, quesadillas, or casseroles.

Do NOT include cheese on pizza.

- ◯ Never
- ◯ 1 time in last month
- ◯ 2-3 times in last month
- ◯ 1 time per week
- ◯ 2 times per week
- ◯ 3-4 times per week
- ◯ 5-6 times per week
- ◯ 1 time per day
- ◯ 2 or more times per day

During the past month, how often did you eat whole grain bread including toast, rolls, and bread in sandwiches?

Whole grain breads include whole wheat, rye, oatmeal and pumpernickel.

Do NOT include white bread.

- ◯ Never
- ◯ 1 time in last month
- ◯ 2-3 times in last month
- ◯ 1 time per week
- ◯ 2 times per week
- ◯ 3-4 times per week
- ◯ 5-6 times per week
- ◯ 1 time per day
- ◯ 2 or more times per day

During the past month, how often did you eat brown rice or other cooked whole grains, such as bulgur, cracked wheat, or millet?

Do NOT include white rice.

- ◯ Never
- ◯ 1 time in last month
- ◯ 2-3 times in last month
- ◯ 1 time per week
- ◯ 2 times per week
- ◯ 3-4 times per week
- ◯ 5-6 times per week
- ◯ 1 time per day
- ◯ 2 or more times per day

During the past month, how often did you eat any chocolate or any other types of candy?

Do NOT include sugar-free candy.

- ◯ Never
- ◯ 1 time in last month
- ◯ 2-3 times in last month
- ◯ 1 time per week
- ◯ 2 times per week
- ◯ 3-4 times per week
- ◯ 5-6 times per week
- ◯ 1 time per day
- ◯ 2 or more times per day

During the past month, how often did you eat doughnuts, sweet rolls, danishes, muffins, pan dulce, or pop-tarts?

Do NOT include sugar-free items.

- ◯ Never
- ◯ 1 time in last month
- ◯ 2-3 times in last month
- ◯ 1 time per week
- ◯ 2 times per week
- ◯ 3-4 times per week
- ◯ 5-6 times per week
- ◯ 1 time per day
- ◯ 2 or more times per day

During the past month, how often did you eat cookies, cake, pie, or brownies?

Do NOT include sugar-free kinds.

- ◯ Never
- ◯ 1 time in last month
- ◯ 2-3 times in last month
- ◯ 1 time per week
- ◯ 2 times per week
- ◯ 3-4 times per week
- ◯ 5-6 times per week
- ◯ 1 time per day
- ◯ 2 or more times per day

During the past month, how often did you eat ice cream or other frozen desserts?<br><br>Do NOT include sugar-free kinds.	◯ Never<br>◯ 1 time in last month<br>◯ 2-3 times in last month<br>◯ 1 time per week<br>◯ 2 times per week<br>◯ 3-4 times per week<br>◯ 5-6 times per week<br>◯ 1 time per day<br>◯ 2 or more times per day
During the past month, how often did you eat popcorn?	◯ Never<br>◯ 1 time in last month<br>◯ 2-3 times in last month<br>◯ 1 time per week<br>◯ 2 times per week<br>◯ 3-4 times per week<br>◯ 5-6 times per week<br>◯ 1 time per day<br>◯ 2 or more times per day

Food Availability The next questions are about the food eaten in your household in the last 12 months and whether you were able to afford the food you need.

Time 9 ____________________

Within the past 12 months, you worried whether your food would run out before you got money to buy more.
◯ Often true
◯ Sometimes true
◯ Never true
◯ Don't know

In the past 12 months, have you received food stamps, also called SNAP, the Supplemental Nutrition Assistance Program on an EBT card?
◯ Yes
◯ No
◯ Don't know

In the next 3 months, on a scale of 1-5 (1 being not at all confident - 5 being 100% confident) how confident are you that you can...?

Eat 5 or more servings of vegetables each day?
◯ 1. Not at all confident
◯ 2
◯ 3
◯ 4
◯ 5. 100% confident

Be physically active at least 30 minutes each day?
◯ 1. Not at all confident
◯ 2
◯ 3
◯ 4
◯ 5. 100% confident

Avoid processed meats and limit red meat intake?
◯ 1. Not at all confident
◯ 2
◯ 3
◯ 4
◯ 5. 100% confident

Limit or avoid alcohol; including avoidance of binge drinking?
Binge drinking is ≥ 4 drinks for women, ≥ 5 drinks for men on the same occasion.
◯ 1. Not at all confident
◯ 2
◯ 3
◯ 4
◯ 5. 100% confident
◯ I don't drink alcohol

Get the HPV vaccination?
◯ 1. Not at all confident
◯ 2
◯ 3
◯ 4
◯ 5. 100% confident
◯ I have completed all recommended doses of the HPV vaccine

Regularly wear sunscreen, hat, protective clothing and use shade between 10am-4pm daily?
◯ 1. Not at all confident
◯ 2
◯ 3
◯ 4
◯ 5. 100% confident

Not use tobacco or nicotine products (cigarettes, vaping/e-cigarettes, smokeless tobacco, etc.)?	◯ 1. Not at all confident<br>◯ 2<br>◯ 3<br>◯ 4<br>◯ 5. 100% confident<br>◯ I don't use nicotine products
Diet Screener + Food Security Time Elapsed	______________________
How much do you agree or disagree with the following statements about the information you hear about cancer?	
There are so many different recommendations about preventing cancer, it's hard to know which ones to follow.	◯ Strongly disagree<br>◯ Disagree<br>◯ Neither agree nor disagree<br>◯ Agree<br>◯ Strongly agree
There is so much cancer information, I don't even care to hear new things about cancer.	◯ Strongly disagree<br>◯ Disagree<br>◯ Neither agree nor disagree<br>◯ Agree<br>◯ Strongly agree
There is so much cancer information, I forget most of it right after I learn it.	◯ Strongly disagree<br>◯ Disagree<br>◯ Neither agree nor disagree<br>◯ Agree<br>◯ Strongly agree
How much do you agree or disagree with the following statements about vaccinations?	
I am completely confident that vaccines are safe.	◯ Strongly disagree<br>◯ Disagree<br>◯ Neither agree nor disagree<br>◯ Agree<br>◯ Strongly agree
Everyday stress prevents me from getting vaccinated.	◯ Strongly disagree<br>◯ Disagree<br>◯ Neither agree nor disagree<br>◯ Agree<br>◯ Strongly agree
When I think about getting vaccinated, I weigh benefits and risk to make the best decision possible.	◯ Strongly disagree<br>◯ Disagree<br>◯ Neither agree nor disagree<br>◯ Agree<br>◯ Strongly agree
Cancer Questions Time Elapsed	______________________
Time 10	______________________

Please read each of the following items carefully, thinking about how it relates to your life, and then indicate how true it is for you.

	1. Not true at all	2.	3.	4.	5. Completely true
I feel a sense of choice and freedom in the things I undertake	○	○	○	○	○
I feel disappointed with many of my performances.	○	○	○	○	○
I feel that people who are important to me are cold and distant towards me.	○	○	○	○	○
Most of the things I do feel like "I have to."	○	○	○	○	○
I feel confident that I can do things well.	○	○	○	○	○
I feel that my decisions reflect what I really want.	○	○	○	○	○
I feel connected with people who care for me, and for whom I care.	○	○	○	○	○
I feel excluded from the group I want to belong to.	○	○	○	○	○
I feel forced to do many things I wouldn't choose to do.	○	○	○	○	○
I feel competent to achieve my goals.	○	○	○	○	○
I experience a warm feeling with the people I spend time with.	○	○	○	○	○
I feel insecure about my abilities.	○	○	○	○	○

Demographics These last questions will be used to divide answers into groups.

Time 11	__________________________
Mediators Time Elapsed	__________________________
What is your age?	__________________________
What is your gender Identity?	◯ Woman<br>◯ Man<br>◯ Transgender Woman/Trans Feminine<br>◯ Transgender Man/Trans Masculine<br>◯ Non-binary/Genderqueer/Gender Fluid<br>◯ Two Spirit<br>◯ Prefer to self-describe<br>◯ Prefer not to say
Please self-describe:	__________________________
Which of the following best represents how you think of yourself?	◯ Gay<br>◯ Lesbian<br>◯ Straight: that is not gay or lesbian, etc.<br>◯ Bisexual<br>◯ None of these describe me and I'd like to see additional options
Are any of these a closer description of how you think of yourself?	◯ Queer<br>◯ Polysexual, omnisexual, sapiosexual, or pansexual<br>◯ Asexual<br>◯ Have not figured out or are in the process of figuring out your sexuality<br>◯ Mostly straight, but sometimes attracted to people of your own sex<br>◯ Do not think of yourself as having sexuality<br>◯ Do not use labels to identify yourself<br>◯ Don't know the answer<br>◯ No, I mean something else<br>◯ Prefer not to say
Please self-describe	__________________________
Are you:	◯ Hispanic/Latino<br>◯ Not Hispanic/Latino
Which of the following groups best describes your race? Select all that apply.	☐ American Indian/Alaska Native<br>☐ Asian<br>☐ Native Hawaiian/Other Pacific Islander<br>☐ Black/African American<br>☐ White<br>☐ More than one of these groups

What is the highest level of education have you completed?	◯ Grade 11 or less<br>◯ High school graduate<br>◯ Trade, technical or vocation education beyond high school<br>◯ Some college - 1 year to 3 years<br>◯ 4-year college degree<br>◯ Postgraduate education
Are you currently in school? If yes, please indicate the level you are enrolled.	◯ I am not in school<br>◯ In high school<br>◯ In community college or technical school<br>◯ In 4-year college/university<br>◯ In graduate or other professional school
Are you currently employed?	◯ Working full time (≥ 40 hours/week)<br>◯ Working part time (< 40 hours)<br>◯ Not working and not looking for work<br>◯ Unemployed and looking for work
What job or business do you work in?	◯ Senior professional/ technical worker (doctor, professor, lawyer, architect, engineer, etc)<br>◯ Junior professional/technical worker (midwife, nurse, teacher, editor, photographer, etc)<br>◯ Administrator/executive/ manager (working proprietor, government official, section chief, department or bureau director, administrative cadre)<br>◯ Office staff (secretary, office worker)<br>◯ Skilled worker (foreman, group leader, craftsman)<br>◯ Non-skilled worker (laborer, construction, yard worker)<br>◯ Police<br>◯ Military<br>◯ Other security (not police or military)<br>◯ Service worker (housekeeper, cook, waiter, hairdresser, retail sales, child care worker)<br>◯ Other<br>◯ Don't know<br>◯ Prefer not to answer
Other job or business category, please specify:	__________________________________
Are you:	◯ Married<br>◯ Partnered but not married<br>◯ Separated, divorced or widowed<br>◯ Single
Who do you regularly live with? (Please select all that apply)	☐ With parent(s)<br>☐ With sibling(s) (brother or sister)<br>☐ With other relatives (grandparents, aunts, uncles, etc.)<br>☐ With spouse/partner<br>☐ With children<br>☐ With friends/roommates<br>☐ I live by myself<br>☐ Other
Other arrangement (Please specify):	__________________________________

Are you a parent of your own children (biological, adopted, or fostered) or stepchildren who are under age 18?	◯ Yes<br>◯ No
Do you live with your own biological or step-children who are under age 18?	◯ Yes<br>◯ No
Generally speaking, do you usually think of yourself as:	◯ Conservative<br>◯ Middle-of-the-road<br>◯ Liberal
Which best describes how frequently you attend church or religious services and activities?	◯ Never<br>◯ Rarely<br>◯ A few times a year<br>◯ At least monthly<br>◯ Weekly or more
What is your birthdate?	______________________________
How tall are you?<br><br>______ feet (e.g. 5 feet)<br>______ inches (e.g. 4 inches)	
About how much do you weigh (in pounds)?	______________________________
BMI	______________________________
Do you have any kind of health care coverage? This includes health insurance, prepaid plans such as preferred provider organization (PPO), health maintenance organization (HMO), point-of-service plan (POS), or high-deductible plan with health savings account (HSA), or government plans such as Medicaid, TRICARE, Veterans Health Administration, or Indian Health Service?	◯ Yes<br>◯ No<br>◯ I don't know
About how long has it been since you last visited a doctor for a routine checkup? A routine checkup is a general physical exam, not an exam for a specific injury, illness, or condition.	◯ Within the past year (anytime less than 12 months ago)<br>◯ Within the past 2 years (1 year but less than 2 years ago)<br>◯ Within the past 5 years (2 years but less than 5 years ago)<br>◯ 5 or more years ago<br>◯ Never
Has a doctor ever told you that you had cancer?	◯ Yes<br>◯ No
Has a doctor ever told someone in your immediate family (parents, brother, sister, spouse, or child) that they had cancer?	◯ Yes<br>◯ No

These are all the questions we have. Thank you for taking part in the survey.

Total Survey Time (minutes) __________________________________
